# Deprescribing Education vs Usual Care for Patients With Cognitive Impairment and Primary Care Clinicians: The OPTIMIZE Pragmatic Cluster Randomized Trial

**DOI:** 10.1001/jamainternmed.2022.0502

**Published:** 2022-03-28

**Authors:** Elizabeth A. Bayliss, Susan M. Shetterly, Melanie L. Drace, Jonathan D. Norton, Mahesh Maiyani, Kathy S. Gleason, Jennifer K. Sawyer, Linda A. Weffald, Ariel R. Green, Emily Reeve, Matthew L. Maciejewski, Orla C. Sheehan, Jennifer L Wolff, Courtney Kraus, Cynthia M. Boyd

**Affiliations:** 1 Institute for Health Research, Kaiser Permanente Colorado, Aurora; 2 Department of Family Medicine, University of Colorado School of Medicine, Aurora; 3 Division of Geriatric Medicine and Gerontology, Johns Hopkins University School of Medicine, Baltimore, Maryland; 4 Department of Clinical Pharmacy, Kaiser Permanente Colorado, Aurora; 5 Quality Use of Medicines and Pharmacy Research Centre, School of Pharmacy and Medical Science, University of South Australia, Adelaide, South Australia, Australia; 6 Durham Center of Innovation to Accelerate Discovery and Practice Transformation (ADAPT), Veterans Affairs Medical Center, Durham, North Carolina; 7 Department of Population Health Sciences, Duke University Medical Center, Durham, North Carolina; 8 School of Public Health, Johns Hopkins School of Medicine, Baltimore, Maryland

## Abstract

**Background:**

Individuals with dementia or mild cognitive impairment frequently have multiple chronic conditions (defined as ≥2 chronic medical conditions) and take multiple medications, increasing their risk for adverse outcomes. Deprescribing (reducing or stopping medications for which potential harms outweigh potential benefits) may decrease their risk of adverse outcomes.

**Objective:**

To examine the effectiveness of increasing patient and clinician awareness about the potential to deprescribe unnecessary or risky medications among patients with dementia or mild cognitive impairment.

**Design, Setting, and Participants:**

This pragmatic, patient-centered, 12-month cluster randomized clinical trial was conducted from April 1, 2019, to March 31, 2020, at 18 primary care clinics in a not-for-profit integrated health care delivery system. The study included 3012 adults aged 65 years or older with dementia or mild cognitive impairment who had 1 or more additional chronic medical conditions and were taking 5 or more long-term medications.

**Interventions:**

An educational brochure and a questionnaire on attitudes toward deprescribing were mailed to patients prior to a primary care visit, clinicians were notified about the mailing, and deprescribing tip sheets were distributed to clinicians at monthly clinic meetings.

**Main Outcomes and Measures:**

The number of prescribed long-term medications and the percentage of individuals prescribed 1 or more potentially inappropriate medications (PIMs). Analysis was performed on an intention-to-treat basis.

**Results:**

This study comprised 1433 individuals (806 women [56.2%]; mean [SD] age, 80.1 [7.2] years) in 9 intervention clinics and 1579 individuals (874 women [55.4%]; mean [SD] age, 79.9 [7.5] years) in 9 control clinics who met the eligibility criteria. At baseline, both groups were prescribed a similar mean (SD) number of long-term medications (7.0 [2.1] in the intervention group and 7.0 [2.2] in the control group), and a similar proportion of individuals in both groups were taking 1 or more PIMs (437 of 1433 individuals [30.5%] in the intervention group and 467 of 1579 individuals [29.6%] in the control group). At 6 months, the adjusted mean number of long-term medications was similar in the intervention and control groups (6.4 [95% CI, 6.3-6.5] vs 6.5 [95% CI, 6.4-6.6]; *P* = .14). The estimated percentages of patients in the intervention and control groups taking 1 or more PIMs were similar (17.8% [95% CI, 15.4%-20.5%] vs 20.9% [95% CI, 18.4%-23.6%]; *P* = .08). In preplanned subgroup analyses, adjusted differences between the intervention and control groups were –0.16 (95% CI, –0.34 to 0.01) for individuals prescribed 7 or more long-term medications at baseline (n = 1434) and –0.03 (95% CI, –0.20 to 0.13) for those prescribed 5 to 6 medications (n = 1578) (*P* = .28 for interaction; *P* = .19 for subgroup interaction for PIMs).

**Conclusions and Relevance:**

This large-scale educational deprescribing intervention for older adults with cognitive impairment taking 5 or more long-term medications and their primary care clinicians demonstrated small effect sizes and did not significantly reduce the number of long-term medications and PIMs. Such interventions should target older adults taking relatively more medications.

**Trial Registration:**

ClinicalTrials.gov Identifier: NCT03984396

## Key Points

**Question:**

Can increasing awareness about deprescribing prior to primary care visits reduce the use of potentially inappropriate long-term medications for individuals with cognitive impairment?

**Findings:**

In this pragmatic cluster randomized clinical trial of deprescribing education for 3012 older adults with cognitive impairment taking 5 or more medications and their primary care clinicians, patients from intervention clinics and control clinics were taking a similar mean number of medications (6.4 vs 6.5) at 6 months, and similar proportions of patients were taking 1 or more potentially inappropriate medications after 6 months.

**Meaning:**

Educating patients and clinicians about deprescribing in primary care did not have an effect on the number of long-term medications or percentage of potentially inappropriate medications for older adults taking 5 or more long-term medications; findings suggest such interventions should target older adults taking relatively more medications.

## Teaser

This cluster randomized clinical trial examines the effectiveness of educating patients and clinicians about the potential to deprescribe unnecessary or risky medications among patients with dementia or mild cognitive impairment.

## Introduction

Individuals with Alzheimer disease and related dementias (ADRD) or mild cognitive impairment (MCI) frequently have multiple chronic medical conditions (defined as ≥2 chronic medical conditions).^1,2^ Individuals with multiple chronic conditions plus cognitive impairment have a greater risk of polypharmacy, potentially inappropriate medications (PIMs), adverse drug events, and further cognitive deterioration and higher rates of hospitalization and mortality than those without impaired cognition.^3,4,5,6,7,8^ Optimizing medications through deprescribing, defined as reducing or stopping medications for which potential harms outweigh potential benefits, can improve outcomes for individuals with multiple chronic conditions, particularly for those with ADRD or MCI.^3,9,10,11,12,13,14^ Prior deprescribing interventions for persons with dementia or cognitive impairment have focused on hospital or skilled nursing settings or on specific medication classes.^15,16,17^

Through extensive stakeholder engagement, we designed a patient-centered, primary care–based pragmatic intervention to educate and engage patients with cognitive impairment, their family members, and primary care clinicians about deprescribing—a multifaceted approach that has been effective for deprescribing interventions.^18,19,20,21,22,23,24,25^ Because more than 90% of older individuals are receptive to discontinuing unnecessary medications when recommended by their clinicians, deprescribing in the context of a primary care relationship may make it easier for clinicians to engage patients and family members in conversations about deprescribing.^26,27,28^ The present study examines the effectiveness of an intervention coupling patient and family deprescribing education and activation (through questions about medication discontinuation) with clinician preparation to encourage discussions of deprescribing across medications in primary care—a recommended approach that has not been previously investigated, to our knowledge.^11,29,30,31^

In a pragmatic cluster randomized clinical trial, the OPTIMIZE (Optimal Medication Management in Alzheimer Disease and Dementia) intervention targeted older adults with ADRD or MCI plus 1 or more additional chronic medical conditions who were prescribed 5 or more long-term medications and their primary care clinicians. The goal of the intervention was to increase patient and clinician awareness about the potential to deprescribe unnecessary or risky medications. We hypothesized that the intervention would reduce the number of long-term medications prescribed and the percentage of individuals taking 1 or more PIMs at 6 months’ follow-up.

## Methods

### Design

The OPTIMIZE intervention was designed to be patient centered and easily incorporated into primary care practice.^18,19,32^ It was conducted as a pragmatic trial at Kaiser Permanente Colorado (KPCO), a not-for-profit integrated health care delivery system. This study was conducted in 18 KPCO adult primary care clinics (9 intervention clinics and 9 control clinics) in the Denver-Boulder geographic area. Clinics were randomized by the study biostatistician using a uniform distribution in blocks of 2 by clinic size to ensure comparable numbers of patients and clinicians. Clinic patient populations ranged from 170 to 1125. The study protocol has been published and was registered on ClinicalTrials.gov (NCT03984396)^18^ (trial protocol in Supplement 1). The KPCO and Johns Hopkins University institutional review boards approved the study. Individual informed consent was waived owing to the pragmatic design, in which all medication decisions were made by primary care clinicians. An institutional review board–approved study information sheet was included with all mailings. This study followed the Consolidated Standards of Reporting Trials (CONSORT) reporting guideline.

### Participants and Setting

The intervention targeted adults aged 65 years or older receiving outpatient care from an established primary care clinician at 1 of the 18 KPCO primary care clinics. Eligibility criteria were health plan enrollment for at least 1 year prior to the intervention, an *International Statistical Classification of Diseases and Related Health Problems, Tenth Revision–*coded diagnosis of ADRD or MCI from any in-person encounter during the preceding year or from the medical record problem list (eTable 1 in Supplement 2), 1 or more additional chronic conditions (from >40 chronic medical conditions listed in the Chronic Conditions Warehouse), and 5 or more long-term medications.^33,34^ Of these individuals, those who scheduled a visit at their primary care clinic at least 7 days in advance were eligible to receive the patient and family component of the intervention. Primary care clinicians providing care for adults in the intervention clinics (family medicine and internal medicine physicians and advanced practice professionals) received the clinician component of the intervention.

### Intervention

The intervention ran from April 1, 2019, to March 31, 2020. It had 2 components: (1) a patient and family component comprising materials mailed in advance of primary care visits and (2) a clinician component of monthly educational materials on deprescribing plus notification in the electronic health record schedule about upcoming visits with participating patients. For the patient intervention, eligible individuals were identified every weekday by study personnel using electronic health record data and visit schedules. Those with upcoming, in-person primary care visits were mailed a brochure (“Managing Medication”) providing information on deprescribing and a 9-question, validated, revised Patients’ Attitudes Toward Deprescribing questionnaire version for cognitive impairment.^18,35^ These materials were intended to educate patients and family members about deprescribing because the education of patients and family members is found to be most effective when coupled with the activation achieved through meaningful questions around specific topics.^36,37,38^ Patients and family members were asked to complete the questionnaire and return it in an included stamped, self-addressed envelope.

Because family members are often engaged in the care of patients with cognitive impairment, the cover letter encouraged patients to share materials with family members. If patients had a second visit more than 8 weeks after their first visit, they received another mailing. Individuals meeting eligibility criteria at control clinics were similarly identified and tracked for outcome measures but did not receive materials.

Clinicians at intervention clinics received a 3-part clinician intervention. First, an initial educational presentation was delivered by the KPCO principal investigator at a monthly clinician meeting. Second, 11 tip sheets and 1 poster on deprescribing topics were handed out (hard copy) to clinicians at monthly clinician meetings. Third, clinicians were notified with an appointment note in the electronic health record (placed by study staff) when they were scheduled to see a patient who had received the intervention brochure. The tip sheets were developed with clinician input, contained suggested language for deprescribing discussions, and addressed high-priority deprescribing topics and situations.^18,19,32^ Clinicians from control clinics did not receive any intervention materials. Usual care at KPCO includes routine medication reconciliation at primary care visits and electronic health record alerts for potentially high-risk prescribing (eg, dose reduction for patients with kidney insufficiency). Patient and family materials and clinician intervention materials are available on the US Deprescribing Research Network website.^39^

### Measures and Data Collection

As a pragmatic trial, variables for eligibility criteria and outcome measurement were designed to be extractable from electronic clinical data. Primary trial outcomes were the number of long-term medications per individual and the percentage of individuals prescribed 1 or more PIMs. These outcomes were selected as reflecting the initial effect of the deprescribing process. We defined long-term medications as any prescribed medication for which the patient received at least a 28-day supply (based on pharmacy dispensing data) excluding selected 2-digit Generic Product Identifier codes (eg, vaccines and local anesthetics; eMethods in Supplement 2). We selected a 28-day supply because long-term opioid medications are often dispensed in 28-day supplies. Counts of medications were defined as the number of long-term medications a patient had on hand based on the number of dispensed days supplied at the time of the baseline clinic visit and 6 months later. Potentially inappropriate medications were based in part on the Beers list of drugs to avoid for individuals with cognitive impairment^40^ plus opioid medications and were defined as an individual having a days’ supply overlapping the identified dates (eTable 2 in Supplement 2). Other analytic variables were extracted from the KPCO Virtual Data Warehouse, a data repository derived from electronic clinical, pharmacy, and administrative data.^41^

### Statistical Analysis

#### Analyses of the Full Cohort

Analysis was performed on an intention-to-treat basis. Primary analyses focused on 6-month follow-up of intervention recipients compared with similar persons at control clinics (N = 3012). We selected 6 months as a sufficient time to permit ongoing discussions between patients, family members, and clinicians about medication discontinuation while minimizing substantial changes in goals of care for most patients. Each individual’s index date was the first clinic appointment eligible for a mailed brochure between March 2019 and early March 2020. Six-month follow-up dates extended through September 2020. We estimated medication counts at 6 months using linear regression analysis of covariance models accounting for baseline medication count and a random clinic effect. Models also included self-reported race and ethnicity, which differed between groups, as well as age and sex. We examined normality of residuals to confirm adequate model fit. The percentage of individuals with 1 or more PIMs was modeled using logistic regression with the same specification.

We initially projected a sample size of 3671, with mean numbers per clinic of approximately 204 and an intraclass correlation coefficient of 0.01.^18^ We estimated that we would be able to detect a decrease in medications of –0.70 or more (ie, <1 medication) with 80% power based on differences of 2 Poisson rates in a cluster randomized design. By the fall of 2019, participant numbers were more than 30% lower than expected primarily owing to a high proportion of clinic appointments scheduled less than 7 days in advance. At that time, we recalculated power for change in medications, conservatively assuming an estimated number of 2590 with a mean number of 145 per clinic. The power remained comparable (–0.70 medications difference detectable) as can occur in cluster randomized trials in which power is driven by the number of clusters, which remained unchanged.^42^ Statistical analysis was conducted with SAS Studio software, release 3.7, enterprise edition (SAS Institute Inc). All *P* values were from 2-sided tests, and results were deemed statistically significant at *P* < .05.

#### Prespecified Additional Analyses

Several prespecified additional analyses of the 2 primary outcomes were conducted. Sensitivity analyses examined whether effect estimates changed when restricting the cohorts to those with 90 days or more of follow-up (ie, excluding those who died or disenrolled from the KPCO health plan prior to 90 days). We also compared subgroups with 5 to 6 long-term medications vs 7 or more long-term medications at baseline, those with ADRD vs MCI, and those who received 1 brochure mailing vs 2 brochure mailings via intervention subgroup interactions. In analysis of covariance models of the medication outcome, an indicator for 7 or more medications would be highly correlated with the baseline medication counts included in the model. Therefore, comparisons of the interactions of 7 or more medications vs 5 to 6 medications by intervention vs delayed control were estimated using constrained repeated-measures models.^43^

Safety monitoring was conducted under guidance of a data safety monitoring board. Safety monitoring compared hospitalization and mortality rates between intervention and control clinics and manually adjudicated medical records for all mortality events and every third hospitalization preceded by a primary care encounter with a medication discontinuation.

## Results

**Figure 1.**
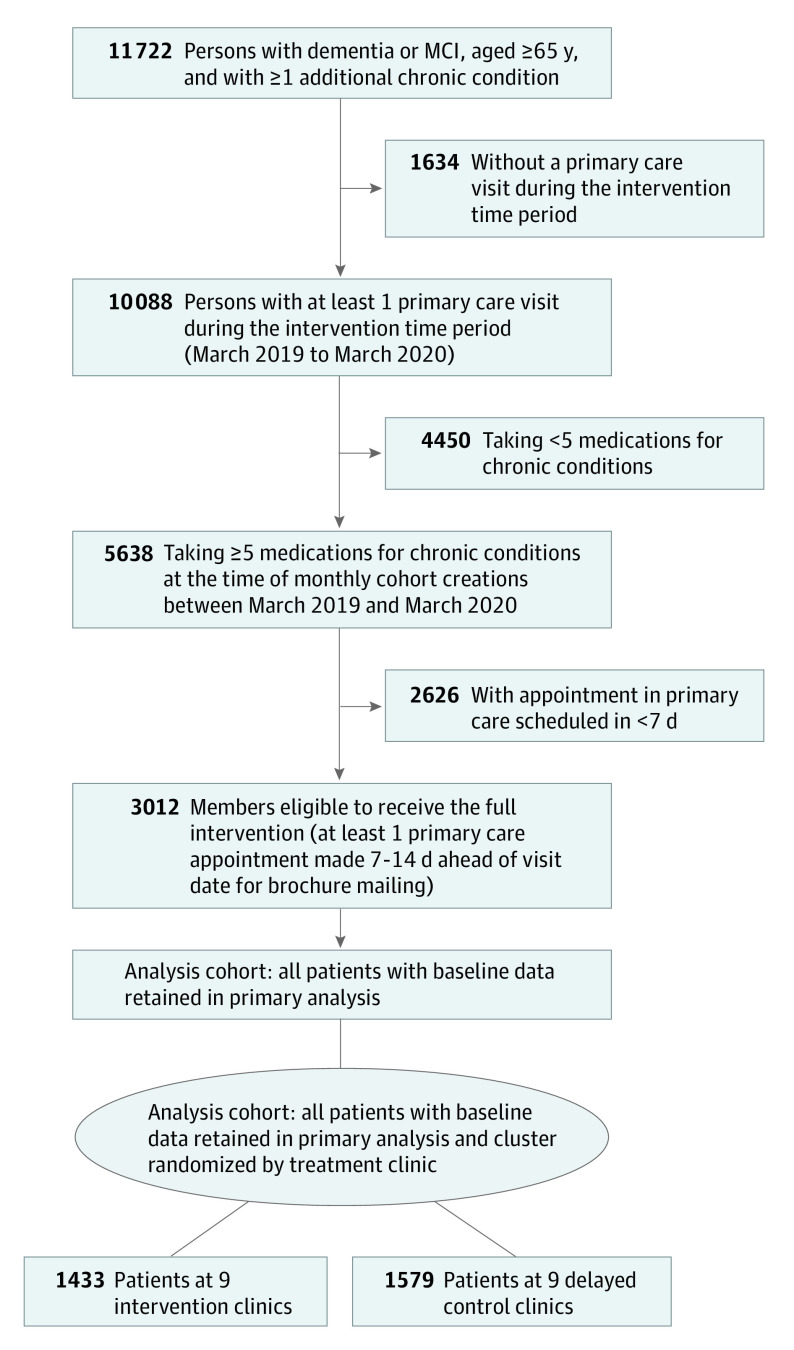
Study Cohort Flow Diagram MCI indicates mild cognitive impairment.

Of 11 722 individuals aged 65 years or older with cognitive impairment and 1 or more additional chronic conditions in the 18 primary care clinics, 3012 met all eligibility criteria (Figure 1). Eligible patients at intervention clinics (n = 1433; 806 women [56.2%]; mean [SD] age, 80.1 [7.2] years) were comparable to those at control clinics (n = 1579; 874 women [55.4%]; mean [SD] age, 79.9 [7.5] years) in mean baseline age, sex, mean (SD) number of long-term medications (7.0 [2.1] in the intervention group and 7.0 [2.2] in the control group), percentage taking 1 or more PIMs (437 of 1433 [30.5%] in the intervention group and 467 of 1579 [29.6%] in the control group), mean (SD) number of chronic conditions (8.5 [3.2] in the intervention group and 8.6 [3.2] in the control group), use of hospice care (23 of 1433 [1.6%] in the intervention group and 33 of 1579 [2.1%] in the control group), death during 6-month follow-up (89 of 1433 [6.2%] in the intervention group and 94 of 1579 [6.0%] in the control group), and disenrollment during 6-month follow-up (31 of 1433 [2.2%] in the intervention group and 29 of 1579 [1.8%] in the control group) (Table 1). More than 91% of individuals were alive and enrolled at 6 months’ follow-up for the intervention (1313 of 1433 [91.6%]) and control (1456 of 1579 [92.2%]) groups.

**Table 1.** Characteristics of the Study Population

Characteristic	Individuals, No. (%)		*P* value^a^
	Intervention (n = 1433 [47.6%])	Control (n = 1579 [52.4%])	
Baseline			
Age, mean (SD), y	80.1 (7.2)	79.9 (7.5)	.48
Female sex	806 (56.2)	874 (55.4)	.62
Race and ethnicity			
Hispanic	118 (8.2)	259 (16.4)	<.001
Non-Hispanic White	1116 (77.9)	1219 (77.2)	
Non-Hispanic, non-White	184 (12.8)	83 (5.3)	
Missing	15 (1.1)	18 (1.1)	
Long-term medications at baseline, mean (SD), No.	7.0 (2.1)	7.0 (2.2)	.83
Percentage with ≥1 PIM at baseline	437 (30.5)	467 (29.6)	.58
Chronic conditions at baseline, mean (SD), No.	8.5 (3.2)	8.6 (3.2)	.32
Mild cognitive impairment diagnosis only	319 (22.3)	342 (21.7)	.69
Received (or eligible for) second brochure mailing	408 (28.5)	484 (30.7)	.19
History of hospice at baseline	23 (1.6)	33 (2.1)	.33
Follow-up			
Died during 6-mo follow-up	89 (6.2)	94 (6.0)	.77
Disenrolled from health plan during 6-mo follow-up	31 (2.2)	29 (1.8)	.52
Hospice during 6-mo follow-up	60 (4.2)	64 (4.1)	.85

Abbreviation: PIM, potentially inappropriate medication.

^a^ *P* = .05 was used as a significance threshold from χ^2^ tests except for age and mean number of long-term medications and conditions at baseline (*t* test).

At 6 months’ follow-up, the estimated mean number of long-term medications was 6.4 (95% CI, 6.3-6.5) in the intervention group vs 6.5 (95% CI, 6.4-6.6) in the control group (adjusted difference, –0.1 [95% CI, –0.2 to 0.04]; *P* = .14) (Table 2). The estimated percentages of patients prescribed 1 or more PIMs were similar between the 2 groups: 17.8% (95% CI, 15.4%-20.5%) in the intervention group vs 20.9% (95% CI, 18.4%-23.6%) in the control group, with an adjusted difference of –3.2 percentage points (95% CI, –6.2 to 0.4 percentage points) (*P* = .08).

**Table 2.** Outcome Estimates and Differences Between Intervention and Control Groups for Number of Long-term Medications and Percentage of Persons With a PIM

Group assignment	Study population, No.	Outcome estimates (95% CI)		
		At 6 mo	Unadjusted difference	Adjusted difference^a^
**Mean long-term medication counts^b^**				
Full cohort (N = 3012)				
Intervention	1433	6.4 (6.3 to 6.5)	–0.1 (–0.2 to 0.03)	–0.1 (–0.2 to 0.04)
Control	1579	6.5 (6.4 to 6.6)		
*P* value	NA	NA	.12	.14
Restricted to persons with ≥90 d of follow-up (n = 2898)				
Intervention	1374	6.4 (6.3 to 6.5)	–0.1 (–0.3 to 0.01)	–0.1 (–0.3 to 0.02)
Control	1524	6.6 (6.5 to 6.7)		
*P* value	NA	NA	.07	.08
**Percentage of persons prescribed ≥1 PIMs^c^**				
Full cohort (N = 3012)				
Intervention	1433	17.8 (15.4 to 20.5)	–3.1 (–6.2 to 0.4)	–3.2 (–6.2 to 0.4)
Control	1579	20.9 (18.4 to 23.6)		
*P* value	NA	NA	.08	.08
Restricted to persons with ≥90 d of follow-up (n = 2898)				
Intervention	1374	17.5 (15.0 to 20.2)	–3.2 (–6.2 to 0.4)	–3.2 (–6.3 to 0.4)
Control	1524	20.7 (18.2 to 23.4)		
*P* value	NA	NA	.08	.08

Abbreviations: NA, not applicable; PIM, potentially inappropriate medication.

^a^ Additionally adjusted for baseline age, sex, and race and ethnicity.

^b^ Long-term medication counts at 6 months and associated intervention minus control differences were estimated using linear regression models adjusted for baseline counts of medications and a random clinic effect.

^c^ Percentage of persons taking a PIM at 6 months and associated intervention minus control differences in logistic regression models adjusted for baseline number of PIMs and a random clinic effect.

**Figure 2.**
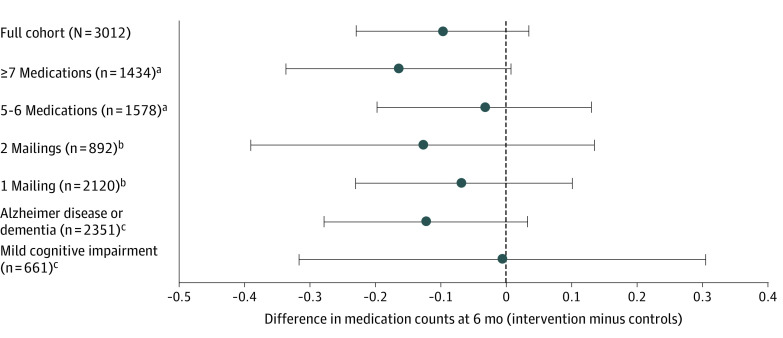
Differences Between Intervention and Control Subgroups in Long-term Medication Counts at 6 Months Estimated differences from linear regression models accounting for baseline counts of medications, age, sex, race and ethnicity, and a random clinic effect. Subgroup models added the appropriate subgroup variable and an interaction with study group. Error bars indicate 95% CIs. ^a^Patients taking 7 or more medications vs 5 to 6 medications (*P* = .28 for interaction). ^b^Two mailings vs 1 mailing (*P* = .70 for interaction). ^c^Alzheimer disease or dementia vs mild cognitive impairment (*P* = .50 for interaction).

**Figure 3.**
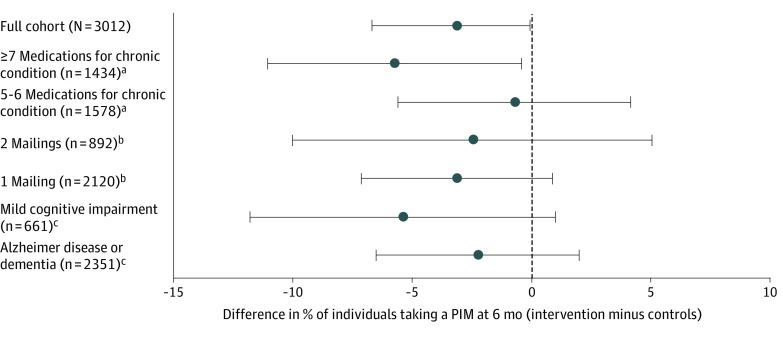
Differences Between Intervention and Control Subgroups in Percentage of Individuals Taking 1 or More Potentially Inappropriate Medications (PIMs) at 6 Months Estimated differences from logistic regression models accounting for baseline PIM, age, sex, race and ethnicity, and a random clinic effect. Subgroup models added the appropriate subgroup variable and an interaction with study group. Error bars indicate 95% CIs. ^a^Patients taking 7 or more medications vs 5 to 6 medications (*P* = .19 for interaction). ^b^Two mailings vs 1 mailing (*P* = .70 for interaction). ^c^Alzheimer disease or dementia vs mild cognitive impairment *(P* = .31 for interaction).

In preplanned sensitivity analyses of cohort members with 90 days or more of follow-up (2898 of 3012), the adjusted estimated mean difference in prescribed long-term medications was –0.1 (95% CI, –0.3 to 0.02) (*P* = .08), and the difference in the percentage of individuals taking 1 or more PIMs was –3.2 percentage points (95% CI, –6.3 to 0.4 percentage points) (*P* = .08) (Table 2). In preplanned subgroup analyses, adjusted differences in long-term medications between intervention and control groups for individuals prescribed 7 or more long-term medications vs 5 to 6 long-term medications were nonsignificant. The adjusted difference was –0.16 (95% CI, –0.34 to 0.01) for the subgroup prescribed 7 or more long-term medications (n = 1434) and –0.03 (95% CI, –0.20 to 0.13) for the subgroup prescribed 5 to 6 medications (n = 1578) (*P* = .28 for interaction) (Figure 2). Among patients taking 5 or 6 long-term medications at baseline, 366 of 1578 (23.2%) had 1 or more PIMs; among those taking 7 or more long-term medications at baseline, 538 of 1434 (37.5%) had 1 or more PIMs. Adjusted differences in the percentages of patients taking 1 or more PIMs for the same 2 subgroups were –5.7 percentage points (95% CI, –11.1 to –0.4 percentage points) for individuals taking 7 or more medications and –0.7 percentage points (95% CI, –5.6 to 4.1 percentage points) for those taking 5 to 6 medications (*P* = .19 for interaction) (Figure 3). There were also no significant adjusted differences in long-term medications or PIMs between the intervention and control groups for individuals who received 1 or 2 brochure mailings or for subgroups with ADRD vs MCI (Figure 2 and Figure 3).

Safety monitoring did not reveal any differences in hospitalization or mortality rates between the intervention or control clinics for individuals in the full cohort during the 4 months after the index date (adjusted risk ratio for hospitalization, 0.92 [95% CI, 0.77-1.09]; adjusted risk ratio for mortality, 1.24 [95% CI, 0.82-1.88]). Record adjudication did not reveal any pattern of adverse drug withdrawal events associated with the intervention.

## Discussion

This large cluster randomized pragmatic intervention of patient, family, and clinician education and activation about deprescribing conducted in 18 primary care clinics did not significantly decrease the number of long-term medications prescribed or the percentage of individuals taking 1 or more PIMs among older adults with cognitive impairment, multiple chronic conditions, and 5 or more long-term medications. There were also no significant interactions between prespecified subgroups. However, these analyses suggest that further investigations should explore whether individuals taking more long-term medications may have a greater potential to benefit from deprescribing. These results have implications for future large-scale pragmatic interventions to decrease the number of unnecessary or risky medications prescribed in vulnerable populations of older adults with cognitive impairment, multimorbidity, and polypharmacy.

Polypharmacy reflects the use of more medications than are medically necessary. Persons with dementia are more likely than noncognitively impaired persons to experience polypharmacy, even accounting for their morbidity burden.^44^ Polypharmacy is often defined as taking 5 or more long-term medications owing to associations with increased risk of medication-related harms above this threshold.^45,46,47^ Accordingly, many deprescribing trials in community populations (including OPTIMIZE) have used this definition to identify target populations.^28,48,49,50,51^ However, other investigations have proposed that individuals prescribed more than 5 medications are at greater risk for adverse effects and are more appropriate targets for deprescribing interventions.^52,53,54,55^ Educational deprescribing interventions, such as OPTIMIZE, may be more fruitful among older adult populations with dementia or cognitive impairment and higher levels of polypharmacy. Individuals taking more long-term medications may have more opportunities for medication refinement and may also be more likely to be prescribed PIMs that would be particularly appropriate for discontinuation. This was true for the OPTIMIZE trial, in which 23% of those taking 5 to 6 long-term medications and 38% of those taking 7 or more long-term medications at baseline had 1 or more PIMs. Deprescribing PIMs is potentially beneficial regardless of total medication burden.^56^

The effect sizes of the OPTIMIZE intervention in the full cohort as well as in prespecified subgroups were small. These effect sizes reflect not only the low-intensity intervention but also the potential for absolute risk reduction of the cohorts and the difference from usual care at KPCO. Other community deprescribing interventions have reported a wide range of effect sizes (0.01-0.95), reflecting a range of approaches and target populations.^11,12,28,55,57^ Given how many older adults experience adverse medication effects resulting in detrimental outcomes, such as admission to a skilled nursing facility, if found to be effective in other investigations, small effect sizes may be acceptable for pragmatic interventions efficiently and safely delivered at scale to targeted populations in primary care.^58,59^

Our primary trial outcomes of number of long-term medications and percentage of individuals taking PIMs reflect the direct effect of deprescribing. Not all medication discontinuations will affect downstream clinical outcomes, such as potentially reduced falls, improved quality of life, or preserved cognition, which are multifactorial and may take longer to manifest. However, reductions in medication burden alone can still be considered a positive outcome of deprescribing if individuals are able to take fewer medications with no detriment to their clinical status.

### Limitations and Strengths

This study had several limitations. Mailing intervention brochures to patients required that appointments be made at least 7 days in advance—an approach selected to avoid interfering with primary care clinic workflow. Although patients and care partners found paper mailings acceptable in the pilot study, this approach was relatively labor intensive and reached only slightly more than half of potentially eligible patients and family members (Figure 1).^18,19^ Future interventions should explore other approaches relevant to different practice settings. Identifying target populations in other settings using data sources relevant to those settings could, for example, assess dementia severity or target caregivers—information that was not available in OPTIMIZE. Although the intervention was delivered during the 12 months ending in March 2020, the follow-up period included the COVID-19 pandemic. Fewer opportunities for clinic visits and deprescribing discussions during the COVID-19 pandemic period in both the intervention and control clinics may have reduced the effect of the intervention on outcomes. However, any pandemic-related care changes likely biased results toward the null. Finally, the list of PIMs was selected for the OPTIMIZE study population and was selected from, and expanded on, the Beers list. We could only identify the medications that were prescribed and dispensed, which excluded over-the-counter medications and likely underascertained PIMs. Any list of PIMs is not a perfect proxy for appropriate medication use, which ultimately reflects clinical need.^40^

This study also has some strengths. A substantial strength of the intervention was the large-scale pragmatic trial design, adding to the generalizability and potential sustainability. All deprescribing decisions and activities were conducted by primary care clinicians and were not made through a deprescribing algorithm or involvement of research personnel. Clinicians were alerted when encountering patients who had been mailed the brochure so that the intervention cohort incorporated clinician activation along with the patient activation—an addition to previous interventions targeting clinicians in which education alone was unlikely to result in substantial deprescribing.^11^ To our knowledge, no published trials have focused on addressing polypharmacy or PIM use in populations with cognitive impairment and multiple chronic conditions in primary care settings.^17^ Finally, large-scale pragmatic interventions may promote subtle, long-lasting outcomes, such as creating a culture of thoughtful deprescribing as part of high-quality care. Focused, intensive deprescribing initiatives within systems already accustomed to deprescribing could produce additional effects in high-priority target populations.

## Conclusions

This pragmatic cluster randomized clinical trial demonstrated that a large-scale patient and clinician education and activation deprescribing intervention can be delivered safely in primary care within an integrated delivery system. The intervention had small effect sizes that did not reach statistical significance; however, subgroup results suggest that similar interventions should be explored in populations with higher levels of polypharmacy. If found to be effective, such approaches could improve downstream clinical deprescribing outcomes and support medication management strategies focused on patient-centered treatment goals.

## Notes

Supplement 1.
Trial Protocol

Supplement 2. **eTable 1.** ICD Codes Used to Identify ADRD and MCI **eMethods.** Long-term Medication Criteria **eTable 2.** Potentially Inappropriate Medications for Patients With Cognitive Impairment Used in OPTIMIZE Intervention

Supplement 3.
Data Sharing Statement
