# Supplementary material for: Deprescribing Education vs Usual Care for Patients With Cognitive Impairment and Primary Care Clinicians: The OPTIMIZE Pragmatic Cluster Randomized Trial
Source: JAMA Intern Med. 2022 Mar 28;182(5):534–42. doi: 10.1001/jamainternmed.2022.0502 (PMC8961395; doi:10.1001/jamainternmed.2022.0502)
Supplement: Supplement 1. — Trial Protocol [file jamainternmed-e220502-s001.pdf]

Optimal Medication Management in Alzheimer's Disease and Dementia

Study Protocol

OPTIMAL MEDICATION MANAGEMENT IN ALZHEIMER'S DISEASE AND DEMENTIA

Co- Principal Investigators:

Elizabeth A. Bayliss, MD, MPSH
Senior Investigator
Institute for Health Research
Kaiser Permanente Colorado (KPCO)

Cynthia M. Boyd, MD, MPH
Professor of Medicine
Division of Geriatric Medicine and Gerontology
Center for Transformative Geriatric Research
Johns Hopkins University School of Medicine

Supported by:

The National Institute on Aging

R33AG057289

TABLE OF CONTENTS **PAGE**

TABLE OF CONTENTS 2
PRÉCIS 4
1 BACKGROUND AND RATIONALE 7
1.1 Background on Condition, Disease, or Other Primary Study Focus 7
1.2 Study Rationale 8
2 STUDY DESIGN 9
2.1 Selection and Enrollment of Participants 12
2.2 Study Enrollment Procedures 13
3 STUDY INTERVENTIONS 13
3.1 Interventions, Administration, and Duration 13
3.2 Delayed Control Intervention 16
3.3 Concomitant Interventions 16
4 STUDY PROCEDURES 16
4.1 Schedule of Evaluations for initial intervention 16
4.2 Description of Evaluations 16
5 SAFETY ASSESSMENTS 19
5.1 Specification of Safety Parameters 20
5.2 Methods and Timing for Assessing, Recording, and Analyzing Safety Parameters 20
5.3 Serious Adverse Events 21
5.4 Reporting procedures 21
5.5 Safety Monitoring 22
6 INTERVENTION DISCONTINUATION 22
7 STATISTICAL CONSIDERATIONS 22
7.1 General Design Issues 22
7.2 Sample Size and Randomization 22
7.2.1 Treatment assignment procedures 23
7.3 Interim analyses and Stopping Rules 23

7.4 Outcomes 24
7.5 Data Analyses 24
8 DATA COLLECTION AND QUALITY ASSURANCE 25
8.1 Data Collection Forms 25
8.2 Data Management 26
8.3 Quality Assurance 26
9 PARTICIPANT RIGHTS AND CONFIDENTIALITY 26
9.1 Human Subjects Materials 26
9.2 Institutional Review Board (IRB) Review 29
9.3 Informed Consent Forms 29
9.4 Participant Confidentiality 30
9.5 Study Discontinuation 30
10 ETHICAL CONSIDERATIONS 30
11 COMMITTEES 31
12 PUBLICATION OF RESEARCH FINDINGS 31
13 REFERENCES 32

PRÉCIS

Study Title

Optimal Medication Management in Alzheimer's Disease and Dementia

Overview and Objectives

The vast majority of individuals with Alzheimer's disease and related dementias (ADRD) as well as those with Mild Cognitive Impairment (MCI) have multiple chronic conditions (MCC). The combination of ADRD plus other conditions (ADRD-MCC) leads to more medication use, more complex medication regimens for patients, and is associated with greater risk of adverse drug events, drug interactions, treatment burden and cognitive changes in addition to higher rates of hospitalization and mortality. Optimizing medications through deprescribing (the process of reducing or stopping the use of inappropriate medications or medications unlikely to be beneficial) can help avoid adverse drug effects and improve outcomes for MCC patients, particularly for those with ADRD or MCI. With input from patients, care partners (family or friends who are involved in care), clinicians, and administrative stakeholders, we have developed a pragmatic deprescribing intervention to educate patients, care partners, and clinicians about deprescribing as one potential element of optimal medication management for ADRD-MCC patients. The intervention will target older adults with ADRD-MCC (and those with MCI plus MCC) on 5 or more medications and their care partners in a pragmatic, cluster-randomized patient- and care partner- centric deprescribing intervention to be conducted at KPCO. For writing clarity, we refer to the study population as those with ADRD-MCC. However, the eligible population includes both ADRD and MCI patients with MCC.

The study objective is to conduct a pragmatic deprescribing intervention for people with ADRD-MCC to test the effectiveness of a primary care based, clinic-level deprescribing intervention on two primary outcomes: number of chronic medications and number of potentially inappropriate medications (PIMs) among seniors with ADRD-MCC.

Hypothesis: A patient (and care-partner)-centered intervention will reduce the number of chronic medications and the number of PIMs among older adults with ADRD-MCC.

Additional study aims address:

Secondary outcomes: Evaluate the effect of the intervention on secondary outcomes collected from clinical data in the EHR: adverse drug events (falls, bleeding episodes, hypoglycemic episodes), reductions in dosage for selected PIMs (benzodiazepines, opioids, antipsychotics), hospital, emergency department and skilled nursing facility utilization, and activities of daily living.

Following the intervention, we will explore mechanisms of intervention effectiveness and uptake through post hoc qualitative interviews with patients, family caregivers, and clinicians; patient and clinician survey responses; and descriptions of outpatient office visit length.

Design and Outcomes

Overview of design

The intervention will be a pragmatic, cluster randomized trial of medication optimization through increased awareness of deprescribing for the ADRD-MCC population. It will be delivered at the clinic level with a delayed control design. As a pragmatic intervention it is designed to be relatively simple, have broad inclusion/exclusion criteria, and be implemented across the KPCO system. The intervention will have two components: a patient/ care partner component focused on education and activation about potential deprescribing, and a clinician component focused on increasing clinician awareness about options and processes for deprescribing in the ADRD-MCC population.

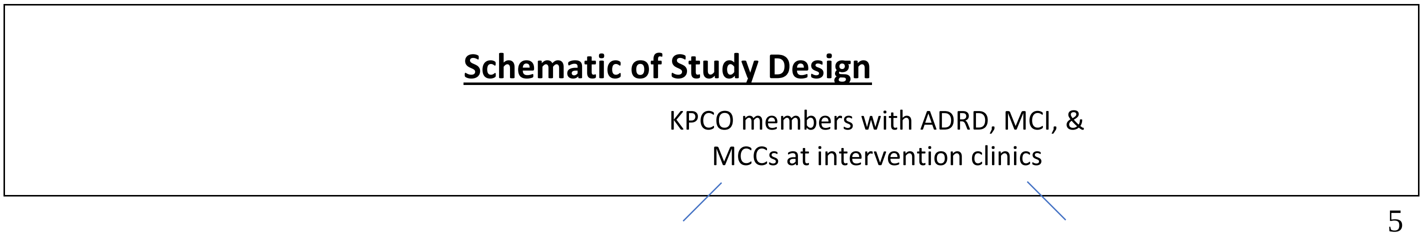

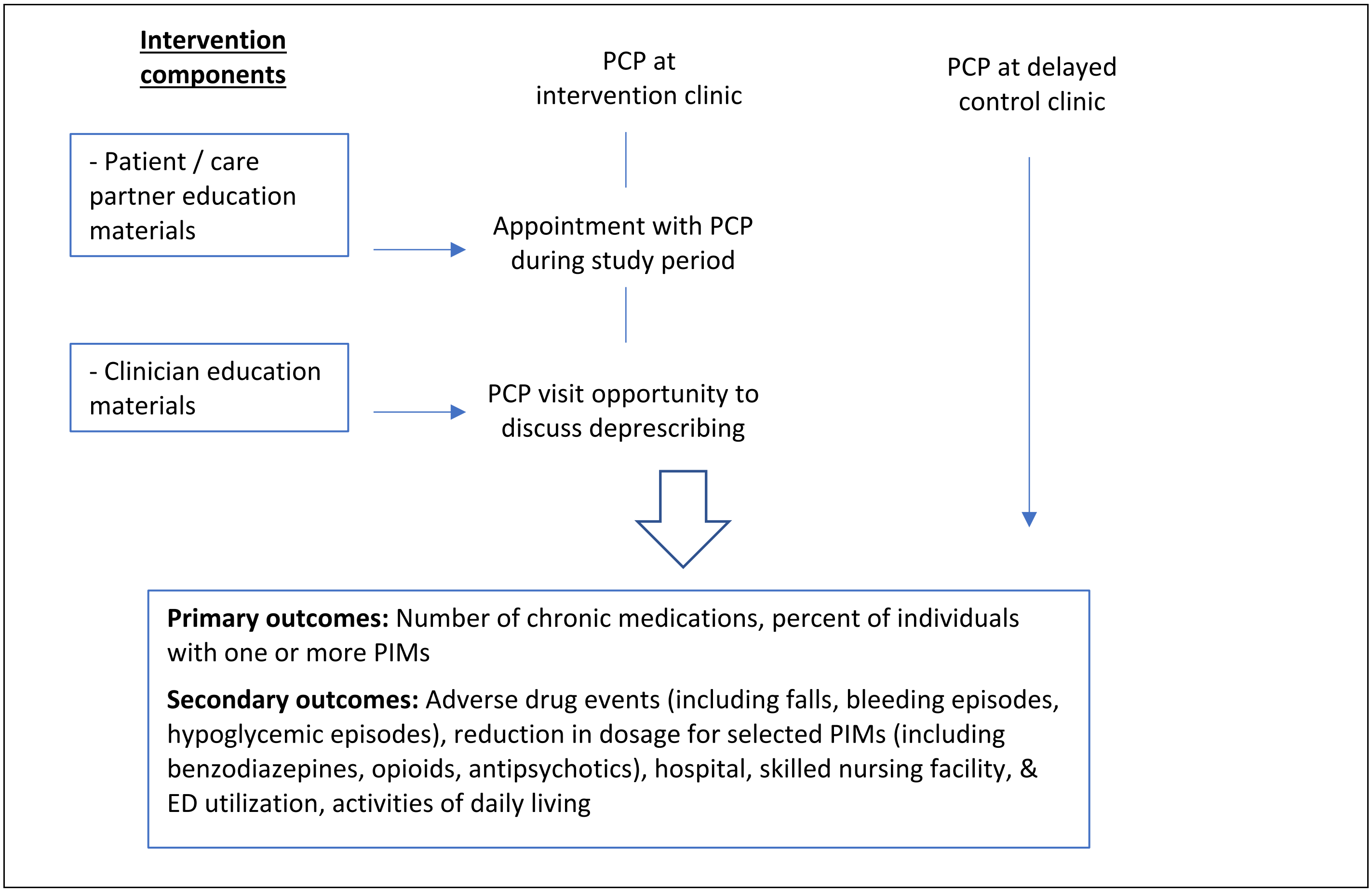

Study Setting

The study will be conducted in the KPCO integrated health care delivery system. KPCO provides healthcare to more than 628,000 members throughout Colorado, including over 75,000 patients with Medicare coverage. The patient population of KPCO is demographically representative of Colorado. KPCO serves patients in 28 medical offices. The intervention will take place at 18 primary care offices in the Denver-Boulder geographic area with 9 as initial intervention sites and 9 as delayed control intervention sites.

Interventions and Duration

There are two components to the intervention: Patient/ care partner education and clinician education. Patient/care partner educational materials about medication optimization will be mailed to eligible members who have upcoming appointments with primary care physicians (PCPs) 7 or more days in advance of scheduled visits. The initial intervention period will run for 12 months (April 2019 to March 2020) and the delayed control intervention period will run for 6 months (~September 2021 to ~February 2022). Primary

care clinicians at the intervention clinics who care for adults (Internal Medicine [IM] and Family Medicine [FM]) will receive education on medication optimization and options for deprescribing through an initial presentation at a monthly team meeting at the beginning of the intervention period, as well as periodic Tip Sheet updates on managing deprescribing in specific situations. PCPs will receive notification via appointment note when materials have been sent to patients with an upcoming appointment.

Sample Size and Study Population

The eligible patient population will be all KPCO members ages 65 or greater with a diagnosis of ADRD or MCI from either ICD visit codes or documentation on the problem list, plus at least one other chronic condition. Patients must also be taking 5 or more chronic medications and have a primary care physician (PCP) at one of the intervention or delayed control clinics. Of this eligible population, those who have at least one visit with a PCP during the intervention period will receive the patient portion of the intervention. The clinician population for the study will be all KPCO primary care physicians who care for adult patients in the Denver Boulder area.

1 BACKGROUND AND RATIONALE

1.1 Background on Condition, Disease, or Other Primary Study Focus

The prevalence of Alzheimer's disease and related dementias (ADRD) increases with age, and is expected to reach 16 million in the U.S. by 2050.[1] The vast majority of individuals with ADRD have multiple chronic conditions (MCC); over 80% of seniors with ADRD have at least one other condition, and 50% have more than 3 other conditions. [2] This morbidity burden associated with ADRD in the U.S. is increasing rapidly.[1,3] For older individuals with ADRD, taking more medications is associated with greater risk of adverse drug events, drug interactions, treatment burden, and cognitive changes from medication side effects.[4] The combination of ADRD plus other conditions (ADRD-MCC) leads to more medication use, more complex medication regimens for patients, and is associated with higher rates of hospitalization and mortality.[1,5,6] Clinical guidelines developed for people with single conditions (e.g. hypertension), rather than for people with MCC (e.g. hypertension and heart disease and dementia and incontinence) tend to exacerbate polypharmacy and complexity of medication regimens and increase the risk of drug interactions and inappropriate prescribing.[7-9] The key principle that *should* guide person- and family-centered care is that people take medicines to help them achieve their goals – but not medicines that are either likely to be harmful or unhelpful.[10]

Although there is considerable information to guide prescribers in the safe and effective initiation of new medications, and additional evidence that deprescribing is itself safe and effective, there is a proportionate lack of guidance regarding how inappropriate medications should be ceased.[11,12] Deprescribing of inappropriate medications (e.g. statins, antihypertensives, and psychotropic medications) in older adults has been shown to be safe, and may lead to beneficial outcomes including improved quality of life, reductions in falls, and improvements in cognitive and psychomotor function.[12-16] Deprescribing is a potentially ideal approach to improving the health and safety of patients with ADRD receiving treatment for comorbid conditions.

Individuals with Mild Cognitive Impairment (MCI), which often precedes dementia, may also benefit from discontinuing or de-intensifying unnecessary or potentially inappropriate medications – especially medications which may have cognitive side effects. For writing clarity, we refer to the study population as those with ADRD-MCC. However, the eligible population will include both ADRD and MCI patients with MCC.

This protocol addresses a pragmatic, primary-care based, cluster randomized trial of deprescribing in older adults with ADRD or MCI and MCC.

1.2 Study Rationale

Optimizing medication use through deprescribing (the process of reducing or stopping the use of inappropriate medications or medications unlikely to be beneficial) can help avoid adverse drug effects and improve outcomes for MCC patients, particularly for those with ADRD.[11,13,17,18] Deprescribing is a nascent field in the U.S. that is likely to have a significant positive impact over the next decade if properly developed and implemented.

Deprescribing interventions in ADRD populations have been largely limited to inpatient or skilled nursing settings or specific medications (e.g. anti-psychotics, statins).[19-22] There is a real need to design interventions that can be targeted at this vulnerable population, integrated into regular practice, and sustained. Interventions that have been effective in reducing the prevalence of PIM use have been multidisciplinary/multifaceted, patient-centered and patient-empowering, and provide ‘direct to consumer’ information. [12,16,23-28] Learnings from these successful interventions include the need to target primary care

clinicians as well as patients, to incorporate patient and family goals, values, and preferences, and to target more than one class of medications--research gaps that our proposed investigation would address.[29,30]

The intervention is designed to enhance both patient information and activation and clinician preparation through a multidimensional approach that is simple to administer at scale. Although physicians have expressed concern that patients and families may be resistant to deprescribing, the literature suggests that this is often not the case, and that fostering patient and family interest in deprescribing may be key to its implementation.[31-50] [11,51,52]

A systematic review of deprescribing interventions found patient-directed educational interventions to be among the most effective, and that involvement and engagement of the primary care provider was also required for success.[28] These are the two elements of our intervention. Patient/caregiver education is most effective when coupled with activation, and activation in turn is achieved through a process of considering and generating personally meaningful questions around current and specific topics.[53-56] Therefore, our patient and care partner materials will include both relevant information delivered to a specific target audience (ADRD-MCC patients at risk for medication adverse effects) and a series of questions to prepare patients and care partners for conversations with their PCPs. Clinician behavior change is more likely to succeed through multidimensional interventions with interactive educational components, on topics relevant to current practice that are implemented on different levels.[57,58] Our intervention components build on this evidence and also expand on it, as there is little information on the effectiveness of coupling patient education and activation with clinician preparation—a novel feature of the intervention.

2 STUDY DESIGN

The study will be a pragmatic, cluster randomized trial with randomization at the level of the clinic and a delayed control group. Clinics will be randomized in blocks of two based on the number of eligible patients per clinic.

Primary outcomes will be total number of medications prescribed and percent of individuals with >1 PIMs prescribed at 6- and 12-months following intervention initiation. Outcomes will be measured with pharmacy dispensing data. PIMs will be based on the Beers List additionally including opioid medications.

Secondary outcomes will include rates of selected adverse drug events (falls, bleeding episodes,

hypoglycemic episodes), dose reductions for selected potentially inappropriate medications (benzodiazepines, opioids, antipsychotics), hospital, emergency department and skilled nursing facility utilization, and activities of daily living reported in the Medicare Health Risk Assessment. Secondary outcomes will be examined following primary outcome analyses.

The eligible patient population will be all KP members ages 65 or greater with a diagnosis of ADRD or MCI from either ICD visit codes or documentation on the problem list, plus at least one other chronic condition. Patients must also be taking 5 or more chronic medications and have a PCP in the KPCO Denver-Boulder area. Of this eligible population, those who schedule a visit with their PCP in an intervention clinic during the study period will receive the intervention.

Exclusion criteria: As the pragmatic trial will be based in primary care clinics, individuals residing in long term care facilities, enrolled in hospice care, or receiving home-based primary care services will be excluded.

The clinician population for the study will be all PCPs who care for adult patients at intervention clinics.

The study setting will be 18 primary care clinics in the KPCO Denver-Boulder geographic area.

The intervention duration will be from April 2019 – February 2022. The initial intervention will run 12 months from March 2019 through April 2020. The intervention for the delayed control group will run from ~September 2021 through ~February 2022. Outcomes for the initial intervention group will be measured at 6 months and 12 months.

Study Timeline

	**Year 1 2019**		**Year 2 2019-2020**				**Year 3 2020-2021**				**Year 4 2021-2022**			
	Mar - May	Jun - Aug	Sep - Nov	Dec - Feb	Mar - May	Jun - Aug	Sep - Nov	Dec - Feb	Mar - May	Jun - Aug	Sep - Nov	Dec - Feb	Mar - May	Jun - Aug
Randomize Clinics	X													
Identify Eligible Participants	X													
Initial Intervention	X	X	X	X										
Delayed										X	X	X		

Intervention														
6-Month Outcome			X	X	X	X								
12-Month Outcome					X	X	X	X						

<u>The patient level intervention</u> consists of materials mailed to all individuals meeting eligibility criteria at intervention clinics. The materials include two components: 1) An informational brochure introducing the idea of discontinuing unnecessary or potentially inappropriate medications as part of optimal medication management. Based on an established model of patient-centered deprescribing and adapted from a direct-to-consumer deprescribing intervention targeting older adults on benzodiazepine medications, this brochure has been revised based on patient and care partner input.[27] 2) A short (9 question), validated version of the revised Patients' Attitudes Towards Deprescribing (rPATD) questionnaire which captures the beliefs, attitudes and experiences of people about deprescribing.[10,51,60-63] The mailing will include a stamped self-addressed envelope to return the rPATD questionnaire. A cover letter for the materials will provide general information about the study along with contact information for the principal investigators and project managers. Completing the rPATD questionnaire serves two purposes for the study: 1) Engaging patients in forming and answering questions is a known method of increasing patient engagement.[54] Additionally, the response data will allow us to explore the effect of patient/care partner attitudes towards deprescribing on outcomes within intervention groups. Materials will encourage patients/ care partners to discuss any interest in deprescribing with their PCP at a primary care visit. The information specifically includes instructions NOT to discontinue any medications without talking to their PCP. Patient/care partner participants will receive materials within a 2-week period prior to a scheduled visit with their PCP. Patients will be eligible for up to two possible mailings if subsequent PCP visits are at least 2 months apart. We will send a second rPATD to patients who have not returned the rPATD from the initial mailing.

<u>The clinician level intervention include</u>s: 1) An initial 15- to 20-minute educational presentation at the clinic department (family medicine or internal medicine) monthly provider meeting focusing on deprescribing as one element of optimal medication management for ADRD individuals. 2) One-page paper Tip Sheets handed out to providers at monthly meetings for 12 months. The Tip Sheets contain information on approaches and language to use in specific medication discontinuation situations. (E.g., One Tip Sheet addresses discussing medications that help with symptoms, but increase the risk of falls such as benzodiazepines, and another Tip Sheet addresses discussing medications for which benefit is unlikely in individuals with limited life expectancy such as statins for primary cardiovascular disease prevention.) The

Tip Sheets were developed based on clinician interviews. 3) PCPs will receive notification via an appointment note in the EHR when materials have been sent to patients with an upcoming appointment. 4) The initial team meeting presentation will include a short (9 question) assessment of Prescribers' Perceptions of Medication Discontinuation.[47,48] This validated instrument contains two domains ('patients' clinical characteristics', and 'clinicians' perceptions of patients' future health') predictive of clinicians' comfort with making discontinuation decisions. As with the patient/ care partner attitudes assessment, this process will serve dual purposes of 1) providing immediate personalized feedback to physicians as education and activation, and 2) providing descriptive information on physician attitudes towards deprescribing. We will collect this information at the initial team meeting. Providers hired during the intervention period will be sent a letter introducing the study, a copy of the Presentation for clinician intervention and the PPMD survey and request that they complete the survey and return it via KPCO inter-department mail. At the end of the intervention, providers will be sent a poster that is a summary of the titles of all Tip Sheets.

2.1 Selection and Enrollment of Participants

Inclusion Criteria:

Patient inclusion criteria are as follows: Age ≥ 65, bonded to a PCP in the KPCO Denver-Boulder geographic area, a diagnosis of ADRD or MCI from ICD-9 or ICD-10 visit codes or from the EHR problem list, one or more additional chronic conditions from a list 86 chronic medical conditions, and be taking 5 or more chronic medications (defined as a 28+ days' supply of medication on the eligibility date; excluding non-indicated medications such as vaccines and anesthetics based on 2-digit GPI codes).

Clinician inclusion criteria: Being a PCP for adult patients at the in the Denver-Boulder geographic area.

Care partner participation: As the intervention is designed to address the needs of the ADRD and MCI population, care partners of patients meeting eligibility criteria may serve as proxy respondents to rPATD materials and as partners during visits with PCPs.

Exclusion Criteria: As the pragmatic trial will be based in primary care clinics, individuals residing in long term care facilities or enrolled in hospice care at baseline will be excluded. We will also exclude individuals residing at home but receiving all their primary care through the Home Rounding service which replaces primary care delivery.

2.2 Study Enrollment Procedures

Intervention recruitment plan. Using EHR data and the KPCO Virtual Data Warehouse (VDW), a quality-controlled common data model derived from multiple KPCO data sources (EHR, membership, pharmacy, laboratory, and other clinical data), study personnel will identify eligible patients who receive primary care at the intervention and control clinics. At least weekly during the intervention period, study personnel will review the list of eligible participants to identify those at an intervention clinic with an appointment with their PCP 7 or more days in advance. Those with upcoming appointments will receive study materials and will be entered into a study database for tracking. Patients will be eligible to receive up to 2 mailings at least 8 weeks apart.

3 STUDY INTERVENTIONS

3.1 Interventions, Administration, and Duration

The intervention will be a pragmatic, cluster randomized trial of medication optimization through increased awareness of deprescribing for the ADRD-MCC and MCI - MCC populations and their primary care clinicians. The trial will be randomized at the clinic level with a delayed control design. As a pragmatic intervention it is designed to be relatively simple, have broad inclusion/exclusion criteria, and be scalable across the KPCO system. The intervention will have two components: a patient/ care partner component focused on education and activation about potential deprescribing, and a clinician component focused on increasing clinician awareness about options and processes for deprescribing in the ADRD-MCC population.

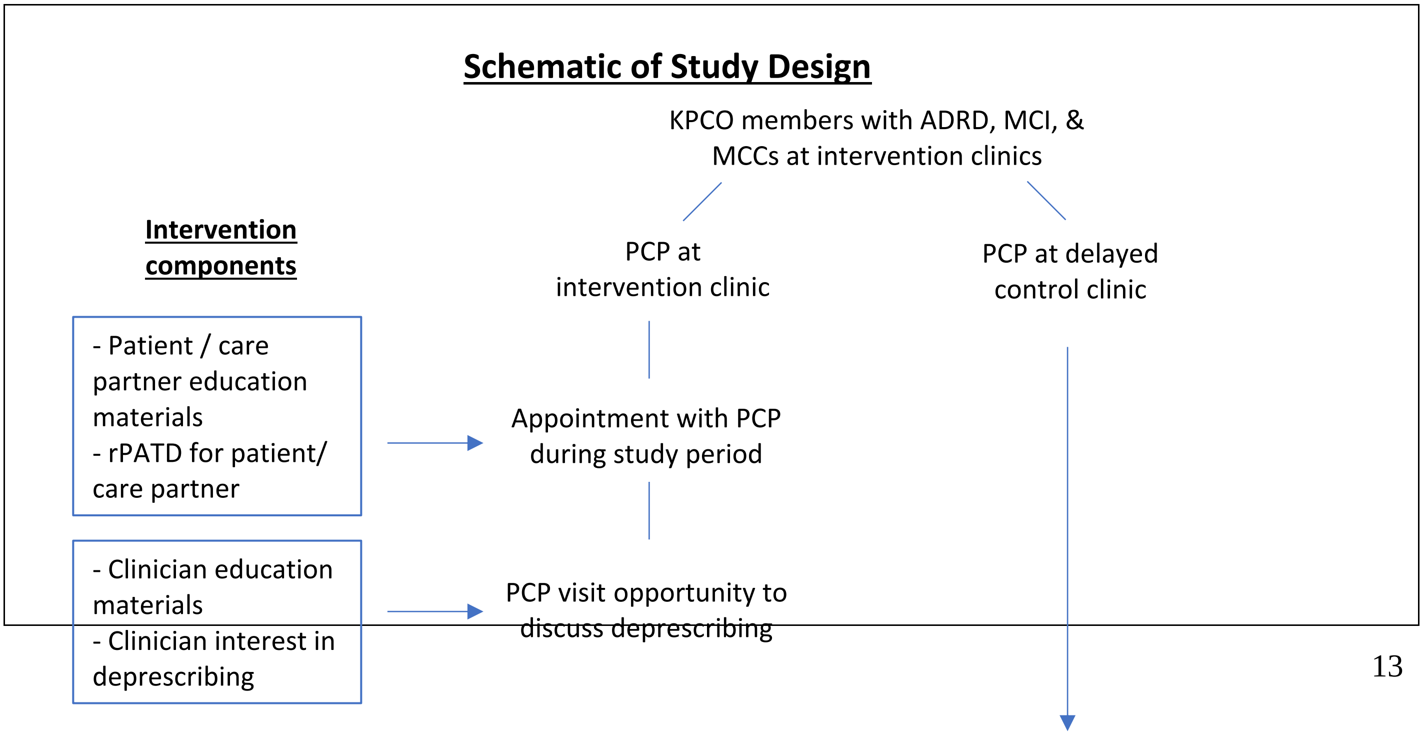

Primary outcomes: Number of chronic medications, percent of individuals with one or more PIMs
Secondary outcomes: Adverse drug events (including falls, bleeding episodes, hypoglycemic episodes), reduction in dosage for selected PIMs (including benzodiazepines, opioids, antipsychotics), hospital, skilled nursing facility, & ED utilization, activities of daily living

Intervention Elements

<u>Initial Intervention</u>

<u>Patient / care partner intervention</u>

- Research analytic staff: Randomize 18 primary care clinics in Denver-Boulder service delivery area to intervention and control using block randomization scheme based on clinic size and number of potentially eligible patients. At least once a week, identify eligible patient population from intervention clinics with ADRD and MCI who have a scheduled appointment 7 or more days in advance.
- Research assistant: Enter identifiers for patients into a tracking database. Individuals with scheduled appointments will receive the following materials:
    - A cover letter providing brief information about the study along with contact information for the principal investigators and project managers. The informational letter will indicate that patients may wish to discuss medication discontinuation with their physician but are under no obligation to do so.
    - A single page tri-fold brochure with educational information on optimizing medicines through considering appropriate medication withdrawal. The brochure includes space for patients/ care partners to list questions should they wish to discuss them with their PCP at their upcoming appointment.
    - The 9-question revised Patients' Attitudes Towards Deprescribing (rPATD) questionnaire, and question about who filled out the questionnaire, along with a stamped and addressed envelope to return questionnaire.
    - Patients will receive a second mailing if they have a second appointment during the intervention period (allowing for a gap of 2 months between appointments).

- At the time of the potential second mailing, a second rPATD will be sent to patients who have not returned the rPATD from the initial mailing.

<u>Clinician intervention</u>

- At the start of the intervention period, the principal investigators and the Project Manager will present a 15-minute educational session to internal medicine and family medicine PCPs at their monthly team meeting at each of the intervention clinics. The presentation will include:
    - A short slide presentation on how deprescribing is one element of optimal medication management for older adults. The presentation will include evidence-based information on what deprescribing is and when and how to consider it. It will also include example situations where deprescribing may be beneficial. The presentation will also provide general information on the study and will include contact information for the study principal investigators and local study staff.
    - We will ask clinicians to complete (on paper) at the meeting a 9-question Prescribers' Perceptions of Medication Discontinuation (PPMD) questionnaire regarding opinions on deprescribing at beginning of presentation.
    - A list of Internal Medicine and Family Medicine clinicians at each of the intervention clinics will be obtained and any providers who missed the initial presentation will be sent a letter of introduction to the study, copy of the Optimize presentation and the PPMD questionnaire, requesting that they complete it and send back to the KPCO Project Manager.
    - Newly hired physicians joining practices will be oriented to the intervention by the KPCO PI and project manager after they complete their initial clinical orientation.
- One-page paper Tip Sheets handed out to providers at monthly meetings. The Tip Sheets contain information on approaches and language to use in specific medication discontinuation situations. Eleven Tip Sheets were developed based on clinician interviews. Example topics include:
    - Limited life expectancy.
    - Potential for harm.
    - Limited evidence for effectiveness.
    - Medications have reached maximum benefit.
- Primary care clinicians will receive notification via an appointment note in the EHR when study materials have been sent to patients with an upcoming appointment.
- At the intervention conclusion, primary care clinicians will be provided a poster that is a colorful

summary of the titles of all Tip Sheets.

3.2 Delayed Control Intervention

We plan to conduct the delayed control intervention in the nine clinics that provided usual care during the initial intervention from ~September 2021 through ~February 2022.

3.3 Concomitant Interventions

There are no concomitant interventions. All medical care will proceed according to the decisions of the patient, care partner and primary care provider.

4 <u>STUDY PROCEDURES</u>

4.1 Schedule of Evaluations for initial intervention

Assessment	Identify Eligible Population	Baseline, Enrollment, 2 weeks prior to scheduled visit	6 months post brochure mailing	12 months post brochure mailing
Inclusion/Exclusion Criteria	X			
Demographics- age, gender, race, ethnicity	X			
Primary outcomes (Aim 1)				
Current Medications	X	X		
Number of Drugs	X	X	X	X
Percent of individuals with one or more Potentially Inappropriate Medications*	X	X	X	X
Secondary outcomes (Aim 2)				
Dose Reductions				X
rPATD		X		
ADE rates		X		X
ADLs		X		X
Hospitalization rate*		X		X
Skilled Nursing Facility admission rate	X	X		X
Mortality*				X
PPMD	X			

* Hospitalization, and mortality will be assessed every 3 months for safety monitoring, but not as secondary outcomes. Medication list attached at end of protocol.

4.2 Description of Evaluations

Study evaluation will focus on primary and secondary quantitative outcomes with initial results focused on primary outcomes.

<u>Definitions of assessment variables from Schedule 4.1 above and associated data elements and sources</u> are itemized

below. Unless otherwise mentioned, all data will be extracted from the KPCO Virtual Data Warehouse, a quality controlled, common data model that includes data from multiple KPCO data sources covering the following domains: Enrollment, utilization, pharmacy, tumor registry, demographics, lab results, mortality, vital signs, census data, geocoded demographic data, patient reported outcomes collected in the course of care, problem list diagnoses, and social history.

Inclusion/ exclusion criteria: as previously described. Data elements will include age, diagnosis codes, location of primary care provider, current medications. Chronic diagnoses will be from a list of 86 chronic conditions itemized in the Multiple Chronic Conditions Chartbook (Gerteis J, et al.). We have developed a method of counting chronic medications that utilizes the Generic Product Index (GPI) in a manner similar to prior work using the WHO Anatomic Therapeutic Chemical index A. Study experts (Boyd, Bayliss, Reeve, Sheehan, Green) created a consensus-based list of GPI categories to be included in estimating the criterion of 5 or more chronic medications (this list excludes drug classes unlikely to be used as chronic medications, such as vaccines and anesthetics).

Demographics: age, gender, self-reported race/ethnicity from the EHR, census-based socioeconomic status.

Diagnoses: ICD-9 (historical) and ICD-10 codes will be used to identify diagnoses from visit billing codes, hospital and emergency department claims, and problem list diagnoses.

Current chronic medications: Total number of chronic medications on the date of study eligibility. Chronic medication use is defined as any medication for which the patient had at least a 28-day supply on the baseline eligibility date. We selected a 28-day supply (rather than a supply for a longer time period) because opioid medications are often dispensed in 28-day supplies. All current medications exclude the following domains identified by 2-digit GPI codes: Vaccines, Toxoids, Allergenic extracts, Oxytocics, Local anesthetics – parenteral, General anesthetics, Antiseptics and disinfectants, Antidotes, Diagnostic products, Chemicals, and Medical devices.

Primary Outcomes:

Number of drugs: number of chronic medications – see above.

Percent of individuals taking one or more PIMs: chronic medications that are listed as potentially inappropriate for older adults based on the Beers list plus opioid medications.

Secondary Outcomes:

Dose reductions: Because calculating dose reductions requires analyzing specific targeted medications, we will assess dose reductions in three selected drug classes. We selected these classes based on the premise that the medications posed risks of adverse side effects, but that patients, care partners, and clinicians may be reluctant to discontinue the medications for fear of recurrent symptoms. The three drug classes are benzodiazepines, opiates, and antipsychotics.

Adverse Drug Events: We will examine three types of ADEs that are common causes of emergency service use among older adults and that may plausibly be expected to decrease in a culture of thoughtful deprescribing. These are falls; hemorrhagic events; and episodes of hypoglycemia in individuals with diabetes. All of these outcomes will be calculated from ICD-10 diagnoses using code lists extracted from the literature and/or previously validated at KPCO. Rates will be calculated for the year prior to baseline and the year following the initial brochure mailing. Denominators will use person-years to account for changes in enrollment and deaths.

ADLs: Activities of Daily Living are reported by patients/ proxy responders as part of the annual Medicare Health Risk Assessment (HRA). These data will be available on the subset of patient participants who have completed the HRA. (Estimated at 45% of the KPCO Medicare beneficiary population.) Data from the most recent HRA will be used.

Hospitalization rate, emergency department visit rate, skilled nursing facility admission rate: Presence/ absence of a one of these admissions over the year prior to baseline and post intervention. (We will distinguish between temporary and permanent admissions to skilled nursing facilities.)

Mortality: Death from any cause during the study period.

Additional planned evaluations:

Following the intervention, we will explore mechanisms of intervention effectiveness and uptake through post hoc qualitative interviews with patients, family caregivers, and clinicians; patient and clinician survey responses; and descriptions of outpatient office visit length. Data and variables include:

rPATD: revised Patients' Attitudes Towards Deprescribing 9-question questionnaire and one question on who completed it. Collected from patients/ care partners as part of the intervention on paper and returned by mail.

PPMD: Physicians Perception of Medication Discontinuation. Collected on paper during the initial portion of the clinician intervention – presentation at clinician team meeting.

Physician specialty: Will be obtained from Colorado Permanente Medical Group data sources.

Time and interval definitions: Enrollment, Baseline, and/or Randomization:
Baseline: The baseline point will be defined as the time at which patients are identified as having an upcoming appointment with their PCP and are mailed the intervention materials. For patients who receive second mailings in advance of a subsequent PCP visit, baseline will remain their first visit but second mailings will be tracked and examined in selected analyses. Baseline for patients in the control clinics will be the date of the first PCP appointment during the initial intervention period. Identical methods will be used to screen control patients for eligibility and identify upcoming visits as described above to ensure comparable identification.

Randomization: Randomization will be at the clinic level and will be stratified by clinic size. (See analytic plan below.)

Follow-up Visits: Data extractions do not depend on follow up visits in this study. Dates for follow up measurements described in the Evaluations Table will be relative to baseline and data will be extracted from the VDW as described above.

5 SAFETY ASSESSMENTS

Safety Monitoring processes were developed in conjunction with the NIA, and the study Data Safety Monitoring Board (DSMB).

5.1 Specification of Safety Parameters

The intervention is educational for both patients / care partners and clinicians and does not in itself actively deprescribe medications. The decision and process for deprescribing is left to the PCP in shared decision making with the patient and his or her care partners.

There is still the potential for adverse effects or unintended consequences from the intervention including: inadvertent stopping of necessary medications by patients or care partners and associated effects of those potential discontinuations, longer patient – PCP visit encounters devoted to medication discussions or clarifying misunderstandings, and lower satisfaction of patients/ care partners about medication management. If PCPs and patient/ care partners elect to discontinue specific medications, there is the potential for recurrent symptoms, adverse drug withdrawal events, and anxiety about the deprescribing process. Potential risks can be minimized or prevented by using a patient-centered, physician-led, structured deprescribing process.

5.2 Methods and Timing for Assessing, Recording, and Analyzing Safety Parameters

There are two primary elements to the safety monitoring plan: comparing rates of potential serious adverse events between intervention and control clinics; and chart abstractions of a sample of patients who experience a potential serious adverse event in each group.

Comparing rates of potential serious adverse events: Every 3 months rates of hospitalizations, and mortality will be compared between groups who have received the intervention (have been sent a mailing) from intervention clinics, and those who would have been eligible to receive the initial mailing from control clinics. These rates will be reported to the DSMB at usual meetings. Chart Abstractions: These will be conducted on all deaths and a sample of every third hospitalization to determine if the potential serious adverse event occurred within 4 months of a medication adjustment by the patient’s primary care physician. Causality related to the medication adjustment will be reviewed in a blinded fashion by steering committee investigators (Reeve, Green, Sheehan). Findings will be reported to the DSMB at usual meetings. Study PIs (Bayliss, Boyd) are blinded to the safety monitoring process which is led by the study biostatistician.

All deaths among individuals who have received the intervention from intervention clinics, and those who would have been eligible to receive the initial mailing from control clinics will be reviewed by a trained Pharm D chart abstractor to determine whether the death occurred within 4 months of a medication

adjustment by the patient's primary care physician. Similarly, every third hospitalization for these two groups will be reviewed to determine the primary diagnosis for that hospitalization and whether the potential serious adverse event occurred within 4 months of a medication adjustment by the patient's primary care physician. (The abstraction sample will be generated weekly.) The PharmD medical record abstractor, a member of the KPCO pharmacy department, will access the tracking database and will review each record using a chart abstraction tool. Records provided to the PharmD will not indicate intervention versus delayed control status but the PharmD cannot be fully blinded due to potential evidence in staff messages related to the study in some charts. In addition to avoiding study status identification on records sent to the PharmD, potential bias will be minimized by detailed chart data extraction steps to be consistently followed.

Throughout the study period any physician, pharmacist or health care staff member will be able to report concerns about a safety issue that arises via an email or phone call to the KPCO Project Manager or the KPCO PI. All clinician Tip Sheets will include contact information for the KPCO PI.

5.3 Serious Adverse Events

Serious adverse events will be defined as hospitalization or mortality. In the ADRD-MCC population we anticipate a baseline hospitalization rate of up to 30% due to morbidity burden.

5.4 Reporting procedures

Follow-up for Adverse Events Reported by Clinic Staff

For any reports from clinic staff of PCPs, the KPCO PI will contact the PCP to review the potential concern and will refer the participant's record for chart abstraction as described above. All potential concerns will be noted and discussed with the DSMB at regular meetings (or immediately for any serious adverse event potentially due to the intervention) and will be reported to the KPCO IRB. Any serious adverse events likely to have resulted from the intervention will be reported to the KPCO IRB, the DSMB and NIA within 5 calendar days. Concerns unlikely to have resulted from the intervention will be summarized for routine DSMB reporting in a separate table prepared by the study biostatistician. Any Serious Adverse Event affecting immediate patient safety will be reported to the KPCO IRB, DSMB, and NIA within 24 hours by

one of the study PIs. Follow-up will extend until discharge from the hospital for hospitalizations or completion of any acute care services associated with an adverse event.

5.5 Safety Monitoring

The Chair of the DSMB will serve as the Safety Officer. DSMB members will not be blinded to intervention groups. DSMB meetings to review safety data will be held at least twice yearly with additional meetings at the discretion of the DSMB chair.

6 INTERVENTION DISCONTINUATION

For this pragmatic intervention, there are no specific intervention discontinuation criteria. The chair of the DSMB will serve as data safety monitor. All clinicians will be able to directly report any safety concerns as described above. Although individual patients/ care partners and clinicians will receive intervention materials, none are required to act on the materials or to participate in deprescribing discussions at any point. This is made explicit in the introductory letter to patients that accompanies intervention materials. Outcomes data will be collected regardless of whether patients / care partners and PCPs in intervention and control clinics choose to discuss medication management or deprescribing.

The KPCO IRB may request study cessation.

7 STATISTICAL CONSIDERATIONS

7.1 General Design Issues

The study design is a pragmatic, primary care-based trial of deprescribing education for patients/ care partners and clinicians which will be randomized at the level of the clinic. The primary hypothesis is that this patient-centered intervention will reduce number of chronic medications and percent of individuals taking one or more PIMs among seniors with ADRD-MCC.

7.2 Sample Size and Randomization

Sample Size and Randomization

There will be 9 intervention clinics and 9 control (delayed intervention clinics). We estimated power using members who would be eligible for the intervention in November 2018. Feasibility data for that cohort showed a median of 5 visits in the prior year with 99% having at least one visit and 80% scheduling a visit at least 7 days in advance. Patient materials are sent in advance of visits only for appointments made at

least 7 days before a visit, therefore, we anticipate approximately 80% of the eligible intervention population will receive mailings during the study period.

We identified 3,671 eligible members with numbers per clinic ranging from 60 to 350. For analyses of counts of medications, we will have > 80% power to detect a decrease of -0.70 (i.e. < 1 medication; power from difference in two Poisson rates in cluster-randomized trial given event rate of 6.8 and intraclass correlation coefficient (ICC)=0.01). We will be examining selected subgroups of interest, for example cohort members receiving a higher dose of the intervention or a restricted cohort with more severe dementia. Both of these comparisons could reasonably retain ~70% of the intervention cohort to contrast to the control cohort and we would be able to detect a decrease of 1.4 medications even if ICC was doubled (i.e. 0.02). Comparisons of proportions of members on a PIM will be able to detect a decrease to 29.0% or less for the intervention clinics compared to the expected rate of 35.8% for the control clinics.

7.2.1 Treatment assignment procedures

The intervention will be a cluster randomized trial delivered at the clinic level with a delayed control design so that all potentially eligible KPCO members will have the opportunity to receive enhanced medication management. Outcomes will be measured at the level of the individual. Clinics will be randomized in blocks of two based on the number of eligible patents. In past experience this has balanced patient characteristics, but we will confirm comparability of the key variables for this study between the intervention and delayed control clinics and consider different blocking schemes if appropriate. Limited remaining differences will be described and adjusted for in statistical analyses.

7.3 Interim analyses and Stopping Rules

Please see discussion of adverse events above. There are currently no statistical criteria that would suspend the intervention.

Every 3 months we will compare rates of hospitalizations and mortality between groups who have received the intervention (have been sent a mailing) from intervention clinics, and those who would have been eligible to receive the initial mailing from control clinics. These rates will be reported to the DSMB.

There will be ongoing safety monitoring using chart abstraction as described above. Any serious adverse events potentially due to the intervention will be reviewed with the KPCO IRB and with the DSMB Chair who will serve as the Safety Officer.

7.4 Outcomes

Primary outcomes are number of chronic medications and percent of individuals on one or more potentially inappropriate medications (PIMS).

Secondary outcomes to be addressed following primary analyses include the number of starts of potentially inappropriate medicines, dose reductions for selected potentially inappropriate medications (benzodiazepines, opiates, and antipsychotics), rates of adverse drug events (falls, hemorrhagic events, and hypoglycemic events), activities of daily living as reported in the Medicare Health Risk Assessment, and skilled nursing facility, hospital, and emergency department admissions.

7.5 Data Analyses

The distributions of all variables, including outcomes, predictors, and covariates, will be examined univariately for data integrity, missing data, and outliers. Outliers will be explored with limited chart review. Data transformation may be conducted as appropriate. We will then assess bivariate associations between independent variables, covariates and primary and secondary outcomes. Multilevel models will be used to account for the clinic level randomization and clinic level intervention as well as adjusting for covariates. The models will include random effects for clinic and fixed effects for intervention versus delayed control and time (baseline or 1-year). We will assess possible nesting of patients within providers; however, most patients see multiple medical doctors, physician assistants and/or nurse practitioners. Change in medication use will be estimated in models that examine medication outcomes at follow-up, adjust for baseline medication use and estimate change by including an interaction term for intervention by time. These models can include baseline patient risk factors as well as provider characteristics to account for imbalanced baseline covariates between clinics. Primary analyses will focus on all members meeting eligibility at either baseline or follow-up time periods (6-months and 1 year) and patient indicators will be included as random effects to identify which patients have information at both baseline and follow up versus baseline or follow up only. Secondary analyses will examine the subset of patients who have both baseline and follow-up data available. We expect these secondary analyses will have comparable conclusions but if differences are seen we will explore potential explanations such as unexpected differential deaths or loss-to-follow-up or whether differences in the patient populations might suggest effect modifiers. For change in number of chronic medications, we expect to use Poisson regression mixed models while for change in the proportion of patients on PIMs, and some secondary outcomes, nonlinear

mixed model techniques will be needed. These models already accommodate data that are missing at random (MAR), which will be adequate for some missing covariate data. For items unlikely to be MAR, such as missing questionnaire responses, we will explore multiple imputation methods to estimate missing scores.[65]

We will examine effects of the intervention within the following prespecified subgroups: 1) members who received a higher dose of the intervention (e.g., brochure and more than one visit), 2) members on higher numbers of medications at baseline (7 or more medications), and 3) individuals with MCI vs. ADRD. We anticipate missing data on at least 50% of the cohort for secondary outcome measures of patient reported outcomes of ADLs and health related quality of life and analyses will use a three-pronged approach. First, we will describe persons with and without these measurements by covariates such as age, health care utilization and co-morbidities. Second, we will use multiple imputation to create replicate datasets with plausible values using estimation models that include covariates and the dependent variables themselves. Because multiple imputation can only address data that are missing at random, i.e. missing data explained by measured covariates, our third step will be sensitivity analyses using methods such as pattern mixture models that aid in examining the consistency of results within groups of patients with similar patterns of missing data.[65]

8 <u>DATA COLLECTION AND QUALITY ASSURANCE</u>

8.1 **Data Collection Forms**

The majority of the study data will be collected from the KPCO VDW as described in the Evaluation section.

Responses from patients/ care partners to the rPATD questionnaire will be anonymously answered on paper and collected by mail. The cover letters for the patient / care partner materials explain the anonymous collection process to patients and invites them to respond if desired.

Responses from clinicians to the PPMD questionnaire will also be anonymous and will be collected on paper at team meetings or if they missed the meeting, study information will be sent along with the survey which we request to be completed and returned via KP inter-department mail.

8.2 Data Management

Unless otherwise mentioned, all data will be extracted from the KPCO Virtual Data Warehouse, a quality controlled, research common data model that includes data from multiple KPCO data sources covering the following domains: Enrollment, utilization, pharmacy, tumor registry, demographics, lab results, mortality, vital signs, census data, geocoded demographic data, patient reported outcomes collected in the course of care (ADLs, quality of life), problem list diagnoses, and social history. Clinician characteristics including demographics, years with the organization, and specialty are available from Colorado Permanente Medical Group databases.

Responses to the rPATD and PPMD questionnaires will be entered into a REDCap database that will also be used to track mailings to patients. Identification will be by study ID which will be linked to medical record number by a separate, password-protected crosswalk. Questionnaire results will be used within the intervention group to assess outcomes as a function of willingness to deprescribe by patients/care partners and clinicians.

Names and other identifiers will be kept in separate locked files. Statistical analyses will be performed on de-identified data; participants will never be individually named. All computerized data will be kept on the secured computers or networks at KPCO. These data will be accessible only to research staff, using confidential usernames and passwords.

8.3 Quality Assurance

No additional quality assurance training, committee, or metrics have been initiated beyond IRB training requirements and usual rigorous data confidentiality standards of KPCO. Please see above and also Participant Confidentiality regarding data quality assurance and management.

9 PARTICIPANT RIGHTS AND CONFIDENTIALITY

9.1 Human Subjects Materials

As a research study in which one or more human subjects are prospectively assigned to one or more interventions to evaluate the effects of those interventions on health related biomedical or behavioral outcomes, the current study meets criteria for a clinical trial. This is a pragmatic, cluster randomized trial with randomization at the clinic level to start at Kaiser Permanente Colorado (KPCO) in 2019. The trial is

co-led by investigators from the KPCO Institute for Health Research and the Johns Hopkins University School of Medicine Center on Aging and Health.

Rationale for studying a potentially vulnerable population

The proposed study addresses medication management in a potentially vulnerable population, individuals with Alzheimer's Disease and Related Dementia (ADRD) and mild cognitive impairment (MCI). For older individuals with ADRD, taking more medications is associated with greater risk of adverse drug events, drug interactions, treatment burden, and cognitive changes from medication side effects. For individuals with MCI, taking potentially inappropriate medications may worsen cognition. The combination of ADRD plus other conditions (ADRD-MCC) leads to more medication use, more complex medication regimens for patients, and is associated with higher rates of hospitalization and mortality. Optimizing medication through deprescribing (the process of reducing or stopping the use of inappropriate medications or medications unlikely to be beneficial) can help avoid adverse drug effects and improve outcomes for MCC patients, particularly for those with ADRD. Therefore, the rationale for studying deprescribing in the potentially vulnerable ADRD and MCI population is that this population is at risk for adverse effects from inappropriate medication use and may attain improved health outcomes from discontinuing unnecessary or inappropriate medications.

Approach to potential risks of the proposed study

Potential risks associated with deprescribing include an adverse drug withdrawal event, return of symptoms, and anxiety about the deprescribing process. However, potential risks can be minimized or prevented by using a patient-centered, physician-led, structured deprescribing process. Additionally, potential risks of deprescribing need to be weighed against potential risks of continuing an inappropriate medication. Deprescribing studies, where the intervention involves withdrawal of medications which have been determined to be inappropriate in the individual (which is what our intervention will involve) have been generally shown to be safe. The proposed intervention involves providing tools (including patient/care partner and clinician education) and opportunities (through patient/ care partner and clinician engagement) for patients, care partners, and physicians to identify medications which may be suitable for withdrawal in an individual and to discuss deprescribing as one component of optimal medication management. The decision to deprescribe a medication will be made by the primary care physician and the patient/care partner through shared decision making. Previous qualitative studies indicate that where there

is a discussion of the reasons why the medication is being recommended for withdrawal and shared decision making about deprescribing, this anxiety is minimal.

Potential benefits of the proposed Research to the subject and others

Clinician guided deprescribing has been shown to be safe, effective, and improve outcomes for older patients. Participants may benefit from learning about potential deprescribing as part of medication management and may benefit from shared decision making with their PCPs regarding medication management.

Importance of Knowledge to be Gained

Information gathered from this research has the potential to improve outcomes for patients with ADRD-MCC. For older individuals with ADRD, taking more medications is associated with greater risk of adverse drug events, drug interactions, treatment burden, and cognitive changes from medication side effects. Educating patients and clinicians on optimal medication management can improve health outcomes. If effective, this simple, scalable, pragmatic study design across multiple KPCO clinics may ultimately be replicated in other healthcare systems and settings.

Data and Safety Monitoring Plan

The study PIs (Bayliss and Boyd) have worked with the National Institute on Aging (NIA) to engage a DSMB to monitor the study and report to the KPCO IRB. The DSMB members comprise individuals with expertise in clinical management of dementia and specific expertise on medication management in the ADRD population: research scientists, physicians and pharmacists not associated with the study. The DSMB will review the study protocol and will monitor the progress and safety of all study participants and oversee the validity and integrity of the data and protocol compliance. The DSMB will not be blinded to study arm and will review interim safety reports and outcomes. The DSMB will advise the NIA regarding issues related to participant safety. Drs. Bayliss and Boyd will attend all DSMB meetings. The DSMB will advise the NIA, and the NIA will have the final decision of whether individual participants or clinics should be removed from the study due to safety concerns.

The DSMB will meet twice yearly and will make recommendations to the NIA. The NIA will determine if modification or cessation of the study protocol will be necessary. DSMB responsibilities will include evaluating study progress, adequacy of recruitment, any (intended or unintended) deviations from study

protocol, interim study analyses and results, performance of individual study sites (clinics), and data quality. Information on these factors and any additional requested information will be summarized and provided to the DSMB in advance of each meeting. Drs. Bayliss and Boyd will be responsible for report to the DSMB interim analyses addressing possible safety outcomes (study terminations, adverse events), and data quality. Consistent with rigorous trial design, the PIs and study staff will be blinded to intervention vs. control groups for interim analyses. The study biostatistician will not be blinded and the chart abstractor will not be blinded.

9.2 Institutional Review Board (IRB) Review

Kaiser Permanente Colorado Institutional Review Board

This study involves human subjects' research which will be undertaken at Kaiser Permanente of Colorado (KPCO). All research conducted KPCO complies with the Department of Health and Human Services requirements for safeguarding the rights and welfare of human subjects, regardless of the source of funding. KPCO has an approved Federal-wide Assurance Compliance filed with the Office for Human Research Protections (OHRP). All human subjects' research undertaken in KPCO is reviewed and approved by the KPCO IRB in accordance with this Assurance. The KPCO IRB also serves as the Research Privacy Board and ensures that the privacy and confidentiality or protected health information is maintained, as required by the Health Insurance Portability and Accountability Act (HIPAA).

9.3 Informed Consent Forms

The pragmatic trial will be conducted at the level of the clinic with 9 intervention clinics and 9 control clinics. Cohort size calculations estimate between 60 and 350 potential participants per clinic.

Summary Table of Consent Processes by Aim

**Aim**	**Activity**	**Target population**	**Requested consent process**
**Aim 1:** In a cluster randomized pragmatic trial, test the effectiveness of a primary care based, clinic-level deprescribing intervention on two primary outcomes: number of chronic medications and number of potentially inappropriate medications (PIMs) among seniors with ADRD-MCC.	Send educational materials to patients (brochure and brief questionnaire).	Patients and care partners. Estimated 60-350 members per clinic. 9 clinics randomized to intervention and 9 to delayed control.	Waiver of informed consent. Mailing contains informational letter about the study. Letter specifies that discussing medications with PCP is optional.
	Educational presentation at department meeting.	Primary care clinicians	Waiver of informed consent. Information on the study presented

	Tip sheets to clinicians at monthly department meetings.	who care for adults.	to clinicians at initial department meeting as part of 15-minute deprescribing presentation. PI contact information on all clinician materials.
**Aim 2**: Evaluate the effect of the intervention on secondary outcomes of adverse drug events (falls, bleeding episodes, hypoglycemic episodes), reductions in dosage for selected PIMs (benzodiazepines, opioids, antipsychotics), hospital, emergency department and skilled nursing facility utilization, and activities of daily living.	Analysis of secondary outcomes.	N/ A	N/A

9.4 Participant Confidentiality

Data management

EHR data: Eligible patient/ care partner participants and clinicians will be identified by the study statistician or analyst using EHR data under a waiver of HIPAA Authorization. The study staff have successfully completed HIPAA and human subjects research trainings as required by KPCO. KPCO will comply with our institutions' policies regarding data sharing, data protection, and data file destruction at the earliest date.

Names and other identifiers will be kept in separate locked files. Statistical analyses will be performed on de-identified data; participants will never be individually named. All computerized data will be kept on the secured computers or networks at KPCO. These data will be accessible only to research staff, using confidential usernames and passwords.

9.5 Study Discontinuation

The study may be discontinued at any time by the IRB, the NIA, the OHRP, the FDA, or other government agencies as part of their duties to ensure that research participants are protected.

10 ETHICAL CONSIDERATIONS

All research conducted KPCO complies with the Department of Health and Human Services requirements for safeguarding the rights and welfare of human subjects, regardless of the source of funding. KPCO has an approved Federal-wide Assurance Compliance filed with the Office for Human Research Protections (OHRP). All human subjects' research undertaken in KPCO is reviewed and approved by the KPCO IRB in accordance with this Assurance. The KPCO IRB also serves as the Research Privacy Board and ensures that the privacy and confidentiality or protected health information is maintained, as required by the Health Insurance Portability and Accountability Act (HIPAA).

11 COMMITTEES

Data Safety Monitoring Board (DSMB):

Elizabeth A. Phelan, MD, MS – Chair of DSMB

Associate Professor, Medicine/Gerontology and Geriatric Medicine
Adjunct Associate Professor, Health Services
Schools of Medicine and Public Health
University of Washington
Harborview Medical Center
325 9th Avenue, Box 359755
Seattle, WA 98104-2499
phelane@medicine.washington.edu

Eduard Vasilevskis, MD, MPH – Hospital Medicine

Chief of Section in Hospital Medicine
Vanderbilt University Medical Center
1215 21st Ave. S.
6006 Medical Center East, NT
Nashville, TN 37232
ed.vasilevskis@vumc.org

Warren B. Bilker, PhD

Professor of Biostatistics
Department of Biostatistics and Epidemiology and Department of Psychiatry
Perelman School of Medicine at the University of Pennsylvania
Blockley Hall, Room 601
423 Guardian Drive
Philadelphia, PA 19104-6021
warren@pennmedicine.upenn.edu

12 PUBLICATION OF RESEARCH FINDINGS

Study results will be disseminated in peer-reviewed publications and will be governed by the policies and procedures developed by the study team.

13 REFERENCES

1. Alzheimer's A. 2015 Alzheimer's disease facts and figures. Alzheimers Dement 2015;11:332-84.
2. Lin PJ, Zhong Y, Fillit HM, Chen E, Neumann PJ. Medicare Expenditures of Individuals with Alzheimer's Disease and Related Dementias or Mild Cognitive Impairment Before and After Diagnosis. J Am Geriatr Soc 2016;64:1549-57.
3. Vassilaki M, Aakre JA, Cha RH, et al. Multimorbidity and Risk of Mild Cognitive Impairment. J Am Geriatr Soc 2015;63:1783-90.
4. Gray SL, Anderson ML, Dublin S, et al. Cumulative use of strong anticholinergics and incident dementia: a prospective cohort study. JAMA Intern Med 2015;175:401-7.
5. Lin PJ, Fillit HM, Cohen JT, Neumann PJ. Potentially avoidable hospitalizations among Medicare beneficiaries with Alzheimer's disease and related disorders. Alzheimers Dement 2013;9:30-8.
6. Patel A, Parikh R, Howell EH, Hsich E, Landers SH, Gorodeski EZ. Mini-cog performance: novel marker of post discharge risk among patients hospitalized for heart failure. Circ Heart Fail 2015;8:8-16.
7. Boyd CM, Darer J, Boult C, Fried LP, Boult L, Wu AW. Clinical practice guidelines and quality of care for older patients with multiple comorbid diseases: implications for pay for performance. JAMA 2005;294:716-24.
8. Gurwitz JH. Polypharmacy: a new paradigm for quality drug therapy in the elderly? Arch Intern Med 2004;164:1957-9.
9. Tinetti ME, Bogardus ST, Jr., Agostini JV. Potential pitfalls of disease-specific guidelines for patients with multiple conditions. N Engl J Med 2004;351:2870-4.
10. Jansen J, Naganathan V, Carter SM, et al. Too much medicine in older people? Deprescribing through shared decision making. BMJ 2016;353:i2893.
11. Reeve E, Shakib S, Hendrix I, Roberts MS, Wiese MD. Review of deprescribing processes and development of an evidence-based, patient-centred deprescribing process. Br J Clin Pharmacol 2014;78:738-47.
12. Iyer S, Naganathan V, McLachlan AJ, Le Couteur DG. Medication withdrawal trials in people aged 65 years and older: a systematic review. Drugs Aging 2008;25:1021-31.
13. Scott IA, Hilmer SN, Reeve E, et al. Reducing inappropriate polypharmacy: the process of deprescribing. JAMA Intern Med 2015;175:827-34.
14. Kutner JS, Blatchford PJ, Taylor DH, Jr., et al. Safety and benefit of discontinuing statin therapy in the setting of advanced, life-limiting illness: a randomized clinical trial. JAMA Intern Med 2015;175:691-700.
15. van der Cammen TJ, Rajkumar C, Onder G, Sterke CS, Petrovic M. Drug cessation in complex older adults: time for action. Age Ageing 2014;43:20-5.
16. Page AT, Clifford RM, Potter K, Schwartz D, Etherton-Beer CD. The feasibility and effect of deprescribing in older adults on mortality and health: a systematic review and meta-analysis. Br J Clin Pharmacol 2016;82:583-623.
17. Ekbom T, Lindholm LH, Oden A, et al. A 5-year prospective, observational study of the withdrawal of antihypertensive treatment in elderly people. J Intern Med 1994;235:581-8.
18. Garcia-Gollarte F, Baleriola-Julvez J, Ferrero-Lopez I, Cuenllas-Diaz A, Cruz-Jentoft AJ. An educational intervention on drug use in nursing homes improves health outcomes resource utilization and reduces inappropriate drug prescription. J Am Med Dir Assoc 2014;15:885-91.
19. Alldred DP, Kennedy MC, Hughes C, Chen TF, Miller P. Interventions to optimise prescribing for older people in care homes. Cochrane Database Syst Rev 2016;2:CD009095.
20. Forsetlund L, Eike MC, Gjerberg E, Vist GE. Effect of interventions to reduce potentially inappropriate use of drugs in nursing homes: a systematic review of randomised controlled trials. BMC Geriatr 2011;11:16.

21. Child A, Clarke A, Fox C, Maidment I. A pharmacy led program to review anti-psychotic prescribing for people with dementia. BMC Psychiatry 2012;12:155.
22. Lang PO, Vogt-Ferrier N, Hasso Y, et al. Interdisciplinary geriatric and psychiatric care reduces potentially inappropriate prescribing in the hospital: interventional study in 150 acutely ill elderly patients with mental and somatic comorbid conditions. J Am Med Dir Assoc 2012;13:406 e1-7.
23. Clyne B, Fitzgerald C, Quinlan A, et al. Interventions to Address Potentially Inappropriate Prescribing in Community-Dwelling Older Adults: A Systematic Review of Randomized Controlled Trials. J Am Geriatr Soc 2016;64:1210-22.
24. Gnjidic D, Le Couteur DG, Kouladjian L, Hilmer SN. Deprescribing trials: methods to reduce polypharmacy and the impact on prescribing and clinical outcomes. Clin Geriatr Med 2012;28:237-53.
25. Campbell NL, Boustani MA. Adverse cognitive effects of medications: turning attention to reversibility. JAMA Intern Med 2015;175:408-9.
26. Sakakibara M, Igarashi A, Takase Y, Kamei H, Nabeshima T. Effects of Prescription Drug Reduction on Quality of Life in Community-Dwelling Patients with Dementia. J Pharm Pharm Sci 2015;18:705-12.
27. Tannenbaum C, Martin P, Tamblyn R, Benedetti A, Ahmed S. Reduction of inappropriate benzodiazepine prescriptions among older adults through direct patient education: the EMPOWER cluster randomized trial. JAMA Intern Med 2014;174:890-8.
28. Ostini R, Jackson C, Hegney D, Tett SE. How is medication prescribing ceased? A systematic review. Med Care 2011;49:24-36.
29. Wolff JL, Boyd CM, Gitlin LN, Bruce ML, Roter DL. Going it together: persistence of older adults' accompaniment to physician visits by a family companion. J Am Geriatr Soc 2012;60:106-12.
30. Wolff JL, Guan Y, Boyd CM, et al. Examining the context and helpfulness of family companion contributions to older adults' primary care visits. Patient Educ Couns 2016.
31. Anderson K, Stowasser D, Freeman C, Scott I. Prescriber barriers and enablers to minimising potentially inappropriate medications in adults: a systematic review and thematic synthesis. BMJ Open 2014;4:e006544.
32. Fried TR, Tinetti ME, Iannone L. Primary care clinicians' experiences with treatment decision making for older persons with multiple conditions. Arch Intern Med 2011;171:75-80.
33. Parr JM, Kavanagh DJ, Young RM, McCafferty K. Views of general practitioners and benzodiazepine users on benzodiazepines: a qualitative analysis. Soc Sci Med 2006;62:1237-49.
34. Cook JM, Marshall R, Masci C, Coyne JC. Physicians' perspectives on prescribing benzodiazepines for older adults: a qualitative study. J Gen Intern Med 2007;22:303-7.
35. Iden KR, Hjorleifsson S, Ruths S. Treatment decisions on antidepressants in nursing homes: a qualitative study. Scand J Prim Health Care 2011;29:252-6.
36. Flick U, Garms-Homolova V, Rohnsch G. "And mostly they have a need for sleeping pills": physicians' views on treatment of sleep disorders with drugs in nursing homes. J Aging Stud 2012;26:484-94.
37. Raghunath AS, Hungin AP, Cornford CS, Featherstone V. Use of proton pump inhibitors: an exploration of the attitudes, knowledge and perceptions of general practitioners. Digestion 2005;72:212-8.
38. Clyne B, Cooper JA, Hughes CM, Fahey T, Smith SM. 'Potentially inappropriate or specifically appropriate?' Qualitative evaluation of general practitioners views on prescribing, polypharmacy and potentially inappropriate prescribing in older people. BMC Fam Pract 2016;17:109.
39. Spinewine A, Swine C, Dhillon S, et al. Appropriateness of use of medicines in elderly inpatients: qualitative study. BMJ 2005;331:935.
40. Clyne B, Bradley MC, Hughes CM, et al. Addressing potentially inappropriate prescribing in older patients: development and pilot study of an intervention in primary care (the OPTI-SCRIPT study). BMC Health Serv Res 2013;13:307.

41. Dickinson R, Knapp P, House AO, et al. Long-term prescribing of antidepressants in the older population: a qualitative study. Br J Gen Pract 2010;60:e144-55.
42. Damestoy N, Collin J, Lalande R. Prescribing psychotropic medication for elderly patients: some physicians' perspectives. CMAJ 1999;161:143-5.
43. Britten N, Brant S, Cairns A, et al. Continued prescribing of inappropriate drugs in general practice. J Clin Pharm Ther 1995;20:199-205.
44. Moen J, Norrgard S, Antonov K, Nilsson JL, Ring L. GPs' perceptions of multiple-medicine use in older patients. J Eval Clin Pract 2010;16:69-75.
45. Schuling J, Gebben H, Veehof LJ, Haaijer-Ruskamp FM. Deprescribing medication in very elderly patients with multimorbidity: the view of Dutch GPs. A qualitative study. BMC Fam Pract 2012;13:56.
46. Anthierens S, Tansens A, Petrovic M, Christiaens T. Qualitative insights into general practitioners views on polypharmacy. BMC Fam Pract 2010;11:65.
47. Linsky A, Simon SR, Marcello TB, Bokhour B. Clinical provider perceptions of proactive medication discontinuation. Am J Manag Care 2015;21:277-83.
48. Linsky A, Simon SR, Stolzmann K, Bokhour BG, Meterko M. Prescribers' perceptions of medication discontinuation: survey instrument development and validation. Am J Manag Care 2016;22:747-54.
49. Linsky A, Simon SR, Stolzmann K, Meterko M. Patient Perceptions of Deprescribing (PPoD): Survey Development and Psychometric Assessment. Med Care 2016.
50. Kouladjian L, Gnjidic D, Reeve E, Chen TF, Hilmer SN. Health Care Practitioners' Perspectives on Deprescribing Anticholinergic and Sedative Medications in Older Adults. Ann Pharmacother 2016;50:625-36.
51. Reeve E, To J, Hendrix I, Shakib S, Roberts MS, Wiese MD. Patient barriers to and enablers of deprescribing: a systematic review. Drugs Aging 2013;30:793-807.
52. Reeve E, Wiese MD, Hendrix I, Roberts MS, Shakib S. People's attitudes, beliefs, and experiences regarding polypharmacy and willingness to Deprescribe. J Am Geriatr Soc 2013;61:1508-14.
53. Wagner EH, Austin BT, Davis C, Hindmarsh M, Schaefer J, Bonomi A. Improving chronic illness care: translating evidence into action. Health Aff (Millwood) 2001;20:64-78.
54. Deen D, Lu WH, Rothstein D, Santana L, Gold MR. Asking questions: the effect of a brief intervention in community health centers on patient activation. Patient Educ Couns 2011;84:257-60.
55. Stange KC, Nutting PA, Miller WL, et al. Defining and measuring the patient-centered medical home. J Gen Intern Med 2010;25:601-12.
56. Wolff JL, Roter DL. Family presence in routine medical visits: a meta-analytical review. Soc Sci Med 2011;72:823-31.
57. Mansouri M, Lockyer J. A meta-analysis of continuing medical education effectiveness. J Contin Educ Health Prof 2007;27:6-15.
58. Grol R, Grimshaw J. From best evidence to best practice: effective implementation of change in patients' care. Lancet 2003;362:1225-30.
59. Shehab N, Lovegrove MC, Geller AI, Rose KO, Weidle NJ, Budnitz DS. US emergency department visits for outpatient adverse drug events, 2013-2014. Jama 2016;316:2115-25.
60. Reeve E, Bell JS, Hilmer SN. Barriers to Optimising Prescribing and Deprescribing in Older Adults with Dementia: A Narrative Review. Curr Clin Pharmacol 2015;10:168-77.
61. Reeve E, Low LF, Hilmer SN. Beliefs and attitudes of older adults and carers about deprescribing of medications: a qualitative focus group study. Br J Gen Pract 2016;66:e552-60.
62. Reeve E, Low LF, Shakib S, Hilmer SN. Development and Validation of the Revised Patients' Attitudes Towards Deprescribing (rPATD) Questionnaire: Versions for Older Adults and Caregivers. Drugs Aging 2016;33:913-28.

63. Reeve E, Shakib S, Hendrix I, Roberts MS, Wiese MD. Development and validation of the patients' attitudes towards deprescribing (PATD) questionnaire. Int J Clin Pharm 2013;35:51-6.
64. Tai-Seale M, Olson CW, Li J, et al. Electronic health record logs indicate that physicians split time evenly between seeing patients and desktop medicine. Health Affairs 2017;36:655-62.
65. Enders CK. Applied missing data analysis: Guilford press; 2010.

Potentially Harmful or Inappropriate Medications in Patients with Dementia

Category	Subcategory	Generic name
H2-receptor antagonists		Cimetidine
		Famotidine
		Nizatidine
		Ranitidine
Anticholinergics	Antidepressants	Desipramine
		Nortriptyline
		Paroxetine
		Amoxapine
		Protriptyline
		Trimipramine Maleate
	Antiemetic	Prochlorperazine
	Antihistamines	Meclizine
		Brompheniramine
		Carbinoxamine
		Chlorphen-PE-PPA-Atropine
		Chlorpheniramine
		Clemastine
		Cyproheptadine
		Dexbrompheniramine
		Dexchlorpheniramine
		Diphenhydramine
		Doxylamine
		Hydroxyzine
		Promethazine
		Triprolidine
	Antimuscarinics	Darifenacin
		Fesoterodine
		Oxybutynin

		Solifenacin

1050

Anticholinergics		Tolterodine
		Trospium
		Atropine
		Dimenhydrinate
		Flavoxate
		Propantheline
		Umeclidinium
	Antispasmodics	Aclidinium Bromide
		Clidinium and Chlordiapoxide
		Dicyclomine
		Homatropine
		Hydrocodone with Homatropine
		Hyoscyamine
		Scopolamine
		Belladonna Alkaloids
		Difenoxin with Atropine
		Diphenoxylate with Atropine
		Methylatropine with Amylase
	Other	Methscopolamine
Antipsychotics		Acepromazine
		Aripiprazole
		Asenapine
		Brexpiprazole
		Cariprazine
		Chlorpromazine
		Chlorprothixene
		Clozapine
		Droperidol
		Fluphenazine Decanoate
		Haloperidol
		Iloperidone
		Loxapine
		Lurasidone
		Mesoridazine Besylate
		Molindone
		Olanzapine

1051

Antipsychotics		Paliperidone Palmitate
		Perphenazine
		Pimozide
		Piperazine
		Quetiapine
		Reserpine
		Risperidone
		Thiethylperazine
		Thioridazine
		Trifluoperazine
		Zipresidone
Benzodiazepine hypnotics		Estazolam
		Flurazepam
		Midazolam
		Quazepam
		Temazepam
		Triazolam
Benzodiazepines		Alprazolam
		Chlordiazepoxide
		Clorazepate
		Diazepam
		Halazepam
		Lorazepam
		Oxazepam
		Prazepam
Nonbenzodiazepine hypnotics		Ergoloid Mesylates
		Eszopiclone
		Isoxsuprine
		Zaleplon
		Zolpidem
Opioids		Alfentanil
		Buprenorphine Patch
		Butorphanol
		Codeine
		Dihydrocodeine

		Hydrocodone
		Hydromorphone
		Levomethadyl
		Meperidine
		Methadone
		Morphine
		Opium
		Oxycodone
		Pentazocine
		Propoxyphene
		Remifentanil
		Sufentanil
		Tapentadol
		Tramadol

Reference:
American Geriatrics Society 2015 Updated Beers Criteria for Potentially Inappropriate Medication Use in Older Adults. *J Am Geriatr Soc, 63*(11), 2227-2246.
