# Supplementary material for: Deprescribing Education vs Usual Care for Patients With Cognitive Impairment and Primary Care Clinicians: The OPTIMIZE Pragmatic Cluster Randomized Trial
Source: JAMA Intern Med. 2022 Mar 28;182(5):534–42. doi: 10.1001/jamainternmed.2022.0502 (PMC8961395; doi:10.1001/jamainternmed.2022.0502)
Supplement: Supplement 2. — eTable 1. ICD Codes Used to Identify ADRD and MCI eMethods. Long-term Medication Criteria eTable 2. Potentially Inappropriate Medications for Patients With Cognitive Impairment Used in OPTIMIZE Intervention [file jamainternmed-e220502-s002.pdf]

Supplementary Online Content

Bayliss EA, Shetterly SM, Drace ML, et al. Deprescribing education vs usual care for patients with cognitive impairment and primary care clinicians: the OPTIMIZE pragmatic cluster randomized trial. *JAMA Intern Med.* Published online March 28, 2022. doi:10.1001/jamainternmed.2022.0502

eTable 1. *ICD* Codes Used to Identify ADRD and MCI
eMethods. Long-term Medication Criteria
eTable 2. Potentially Inappropriate Medications for Patients With Cognitive Impairment Used in OPTIMIZE Intervention

This supplementary material has been provided by the authors to give readers additional information about their work.

© 2022 Bayliss EA et al. *JAMA Internal Medicine*.

eTable 1. *ICD* Codes Used to Identify ADRD and MCI

ICD	Code	Category*	Definition
09	290	ADRD	Dementias
09	290.0	ADRD	Senile dementia, uncomplicated
09	290.1	ADRD	Presenile dementia
09	290.10	ADRD	Presenile dementia, uncomplicated
09	290.11	ADRD	Presenile dementia with delirium
09	290.12	ADRD	Presenile dementia with delusional features
09	290.13	ADRD	Presenile dementia with depressive features
09	290.2	ADRD	Senile dementia with delusional or depressive features
09	290.20	ADRD	Senile dementia with delusional features
09	290.21	ADRD	Senile dementia with depressive features
09	290.3	ADRD	Senile dementia with delirium
09	290.4	ADRD	Vascular dementia
09	290.40	ADRD	Vascular dementia, uncomplicated
09	290.41	ADRD	Vascular dementia, with delirium
09	290.42	ADRD	Vascular dementia, with delusions
09	290.43	ADRD	Vascular dementia, with depressed mood
09	290.8	ADRD	Other specified senile psychotic conditions
09	290.9	ADRD	Unspecified senile psychotic condition
09	294.0	ADRD	Amnestic disorder in conditions classified elsewhere
09	294.1	ADRD	Dementia in conditions classified elsewhere
09	294.10	ADRD	Dementia in conditions classified elsewhere without behavioral disturbance
09	294.11	ADRD	Dementia in conditions classified elsewhere with behavioral disturbance
09	294.2	ADRD	Dementia, unspecified, without behavioral disturbance
09	294.21	ADRD	Dementia, unspecified, with behavioral disturbance
09	294.8	ADRD	Other persistent mental disorders due to conditions classified elsewhere
09	310.89	MCI	Mild memory disturbances, not amounting to dementia, associated with senile brain disease –*Other specified nonpsychotic mental disorders following organic brain damage.*
09	331	ADRD	Other cerebral degenerations
09	331.0	ADRD	Alzheimer's disease
09	331.1	ADRD	Frontotemporal dementia
09	331.11	ADRD	Picks disease
09	331.19	ADRD	Other frontotemporal dementia
09	331.2	ADRD	Senile degeneration of brain
09	331.8	ADRD	Other cerebral degeneration
09	331.82	ADRD	Dementia with Lewy bodies
09	331.83	MCI	Mild cognitive impairment
09	331.89	ADRD	Other cerebral degeneration
09	331.9	ADRD	Cerebral degeneration, unspecified
09	780.93	MCI	Memory loss
09	797	ADRD	Senility without psychosis

© 2022 Bayliss EA et al. *JAMA Internal Medicine*.

09	799.52	MCI	Cognitive Communication deficit
10	F01.50	ADRD	Vascular dementia without behavioral disturbance
10	F01.51	ADRD	Vascular dementia with behavioral disturbance
10	F02.80	ADRD	Dementia in other diseases classified elsewhere without behavioral disturbance
10	F02.81	ADRD	Dementia in other diseases classified elsewhere with behavioral disturbance
10	F03.90	ADRD	Unspecified dementia without behavioral disturbance
10	F03.91	ADRD	Unspecified dementia with behavioral disturbance
10	F03.92	ADRD	Presenile with delusional features
10	F03.93	ADRD	Presenile with depressive features
10	F06.8	ADRD	Other specified mental disorders due to known physiological condition
10	F07.89	MCI	Mild memory disturbances, not amounting to dementia, associated with senile brain disease. *-Other personality and behavioral disorders due to known physiological condition.*
10	F19.97	ADRD	Other psychoactive substance use, unspecified with psychoactive substance-induced persisting dementia
10	G30.0	ADRD	Alzheimer’s disease with early onset
10	G30.1	ADRD	Alzheimer’s disease with late onset
10	G30.8	ADRD	Other Alzheimer’s disease
10	G30.9	ADRD	Alzheimer’s disease, unspecified
10	G31.01	ADRD	Picks disease
10	G31.09	ADRD	Other frontotemporal dementia
10	G31.1	ADRD	Senile degeneration of brain, not elsewhere classified
10	G31.83	ADRD	Dementia with Lewy bodies
10	G31.84	MCI	Mild cognitive impairment
10	G31.89	ADRD	Other specified degenerative diseases of nervous system
10	G31.9	ADRD	Degenerative disease of nervous system, unspecified
10	R41.3	MCI	Other amnesia
10	R41.81	MCI	Age-related cognitive decline
10	R41.841	MCI	Cognitive communication deficit

*category abbreviations: ADRD= Alzheimer’s disease and related dementias
MCI=Mild cognitive impairment

List has been previously applied in Green AR, Reifler LM, Bayliss EA, Weffald LA, Boyd CM. Drugs Contributing to Anticholinergic Burden and Risk of Fall or Fall-Related Injury among Older Adults with Mild Cognitive Impairment, Dementia and Multiple Chronic Conditions: A Retrospective Cohort Study. Drugs & aging. 2019;36(3):289-297.

© 2022 Bayliss EA et al. *JAMA Internal Medicine*.

eMethods. Long-term Medication Criteria

Long-term medications were defined by selected GPI codes <u>and</u> a $\geq$ 28-days' supply of dispensed medication.

Long-term medications exclude the following domains identified by 2-digit GPI codes:

Vaccines, Toxoids, Allergenic extracts, Oxytocics, Local anesthetics – parenteral, General anesthetics, Antiseptics and disinfectants, Antidotes, Diagnostic products, Chemicals, and Medical devices.

© 2022 Bayliss EA et al. *JAMA Internal Medicine*.

**eTable 2.** Potentially Inappropriate Medications for Patients With Cognitive Impairment Used in OPTIMIZE Intervention*			
**Category**	**Subcategory**	**Generic Name**	**Number of persons with 1+ in full cohort**
Anticholinergics	Antidepressants	AMOXAPINE	0
		DESIPRAMINE	6
		NORTRIPTYLINE	53
		PAROXETINE	20
		PROTRIPTYLINE	0
		TRIMIPRAMINE	0
	Antihistamines	BROMPHENIRAMINE	0
		CARBINOXAMINE	0
		CHLORPHENIRAMINE	0
		CLEMASTINE	0
		CYPROHEPTADINE	4
		DIMENHYDRINATE	0
		DIPHENHYDRAMINE	0
		DOXYLAMINE	0
		HYDROXYZINE	32
		MECLIZINE	7
		PROMETHAZINE	11
		TRIPROLIDINE	0
	Antimuscarinics	DARIFENACIN	8
		FESOTERODINE	0
		FLAVOXATE	2
		OXYBUTYNIN	49
		SOLIFENACIN	1
		TOLTERODINE	5
		TROSPIUM	589

© 2022 Bayliss EA et al. *JAMA Internal Medicine*.

	Antispasmodics	ATROPINE	46
		BELLADONNA	0
		CLIDINIUM	1
		DICYCLOMINE	65
		HYOSCYAMINE	3
		ISOPROPAMIDE/PROCHLORPERAZINE	0
		PROCHLORPERAZINE	30
		PROPANTHELINE	0
		SCOPOLAMINE	10
	Antipsychotics	ARIPIPRAZOLE	47
		CHLORPROMAZINE	0
		CLOZAPINE	21
		FLUPHENAZINE	5
		HALOPERIDOL	5
		LOXAPINE	14
		OLANZAPINE	206
		PERPHENAZINE	19
		QUETIAPINE	670
		RESERPINE	0
		RESERPINE/HYDROCHLOROTHIAZIDE	0
		RISPERIDONE	161
		THIORIDAZINE	3
		THIOTHIXENE	0
		TRIFLUOPERAZINE	0
		ZIPRASIDONE	16
Benzodiazepine hypnotics		ESTAZOLAM	0
		FLURAZEPAM	0
		QUAZEPAM	0
		TEMAZEPAM	21
		TRIAZOLAM	12
Benzodiazepines		ALPRAZOLAM	76
		CHLORDIAZEPOXIDE	4

© 2022 Bayliss EA et al. *JAMA Internal Medicine*.

		CLONAZEPAM	203
		CLORAZEPATE DIPOTASSIUM	1
		DIAZEPAM	29
		LORAZEPAM	208
		OXAZEPAM	13
H2-receptor antagonists		CIMETIDINE	0
		FAMOTIDINE	309
		RANITIDINE	362
Nonbenzodiazepine hypnotics		ERGOLOID MESYLATES	0
		ESZOPICLONE	0
		ISOXSUPRINE	0
		ZALEPLON	0
		ZOLPIDEM	30
		BUTORPHANOL	8
Opioids		CODEINE	75
		DIHYDROCODEINE	0
		FENTANYL	0
		FENTANYL LA	59
		FENTANYL SA	0
		HYDROCODONE	890
		HYDROMORPHONE	65
		METHADONE	60
		MORPHINE LA	368
		MORPHINE SA	49
		OXYCODONE	7
		OXYCODONE LA	58
		OXYCODONE SA	1245
		TAPENTADOL LA	2

The list of non-opioid PIMs used in OPTIMIZE was based in part on the Beers list of medications to be avoided in individuals with cognitive impairment. American Geriatrics Society 2015 Updated Beers Criteria for Potentially Inappropriate Medication Use in Older Adults. (American Geriatrics Society 2015 Beers Criteria Update Expert Panel, et al. "American Geriatrics Society 2015 updated beers criteria for potentially inappropriate medication use in older adults." Journal of the American Geriatrics Society

© 2022 Bayliss EA et al. *JAMA Internal Medicine*.

63.11 (2015): 2227-2246.) The OPTIMIZE PIM list both selected from and expanded on the Beers list. Other investigations should use lists of PIMs relevant to the specific investigation.

© 2022 Bayliss EA et al. *JAMA Internal Medicine*.
