# Supplementary material for: Deprescribing Education vs Usual Care for Patients With Cognitive Impairment and Primary Care Clinicians: The OPTIMIZE Pragmatic Cluster Randomized Trial
Source: JAMA Intern Med. 2022 Mar 28;182(5):534–42. doi: 10.1001/jamainternmed.2022.0502 (PMC8961395; doi:10.1001/jamainternmed.2022.0502)
Supplement: Supplement 3. — Data Sharing Statement [file jamainternmed-e220502-s003.pdf]

Data Sharing Statement

Bayliss. Deprescribing Education vs Usual Care for Patients With Cognitive Impairment and Primary Care Clinicians. *JAMA Intern Med*. Published March 28, 2022. doi:10.1001/jamainternmed.2022.0502

Data
Data available: No
